# Recent Advances in Biopesticide Research and Development with a Focus on Microbials

**DOI:** 10.12688/f1000research.154392.4

**Published:** 2025-02-10

**Authors:** Kahsay Tadesse Mawcha, Lawrence Malinga, Debbie Muir, Jing Ge, Dennis Ndolo

**Affiliations:** 1 Department of Plant Protection, Hebei Agricultural University, Lingyusi Street, Hebei, 071001, China; 2 International Centre for Genetic Engineering and Biotechnology, Cape Town, South Africa; 3 Department of Plant Sciences, Aksum University, Aksum University, Aksum, Tigray, 1000, Ethiopia; 4 School of Life Sciences, University of KwaZulu-Natal, Pietermaritzburg, South Africa; 5 Crop Protection, South African Sugarcane Research Institute, Durban, South Africa; 6 Department of Forestry, Fisheries and the Environment, Ministry of Enironment, Cape Town, South Africa; 7 Jiangsu Academy of Agricultural Sciences, 50 Zhongling Street, Jiangsu, 210014, China

**Keywords:** biocontrol, bioprotection, formulation, climate-smart agriculture, sustainable pest management

## Abstract

Biopesticides are pest control products derived from natural sources such as microbes, macro-organisms (insects and pathogens), plant extracts, and certain minerals. Many biopesticides are considered environmentally safe and can complement or substitute conventional chemical pesticides. They can also be highly specific or broad spectrum with a unique mode of action controlling a wide range of pest species. Due to their target-specificity and low to no environmental residuality, biopesticides conform to the 3 pillars of Climate-Smart Agriculture, the Sustainable Development Goals, and, ultimately, the Paris Agreement. This review focuses largely on microbial biopesticides derived from fungi, bacteria, viruses, and nematodes. It discusses (i) the various microbial biopesticide formulations, (ii) the mode of microbial biopesticide action, (iii) the factors that affect the potential efficacy of biopesticides, (iv) challenges to the adoption of microbial biopesticides, and (v) the role of microbial biopesticides in Integrated Pest Management programs. Finally, advancements in application techniques, as well as future research directions and gaps, are highlighted.

## 1. Introduction

Biopesticides are naturally derived pest control compounds that offer a relatively safer and target-specific complement to conventional chemical pesticides ( Leo and Rathore 2015; Tijjani et al. 2016; Nuruzzaman et al. 2019; Temiz 2020; Wattimena and Latumahina 2021; Huang et al. 2022). Biopesticides include 1) biochemicals (plant extracts, semiochemicals, microbial extracts/fermentation products, insect growth regulators, compounds synthesized by other organisms, and inorganic compounds), 2) microbial biopesticides (bacteria, fungi, protozoa, viruses, oomycetes, yeast, and algae), 3) macrobials (insect predators, parasitoids, and entomopathogenic nematodes) and classical biocontrol agents ( Copping and Leonard 2000; Chandler et al. 2008; Leahy et al. 2014; Liu et al. 2021; Ram and Singh 2021). Biopesticides play a crucial role in promoting climate-smart agriculture ( CSA) by offering effective pest management solutions, improving crop yield and quality, and reducing pest resistance while minimizing negative impacts on human health and the environment ( Szewczyk et al. 2006; Satish et al. 2017; Kumari et al. 2022). The growing need for biopesticides is partly a consequence of the decrease in the use of conventional pesticides due to farmers increasingly adopting climate-smart agricultural practices and reducing their carbon footprint ( FAO 2019). This shift is driven by several advantages associated with biopesticides, including 1) their ability to slow down the development of pest resistance, 2) low to no toxicity to humans and the environment, hence safeguarding biodiversity, preserving soil health and enhancing food security, 3) their ability to complement chemical pesticides, 4) their low environmental fate, 5) host specificity, 6) biodegradability, 7) minimal risk of post-harvest contamination, 8) resilience against environmental stresses, and 9) compatibility with IPM practices ( Deravel et al. 2014; Kalpana 2021). Biopesticides may, however, not be equally effective against all pests, and their efficacy can vary depending on the target species. Additionally, some pests may have natural resistance or tolerance to certain biopesticides ( Isman 2006).

The excessive reliance on conventional chemical pesticides in agriculture poses a significant threat to environmental health and sustainability, as the agriculture sector contributes a third of the total amount of greenhouse gas emissions. Climate-smart agriculture (CSA) was developed as a framework to contribute to achieving the Sustainable Development Goals (SDGs) under an uncertain climate. Agricultural production systems need to tackle three challenges simultaneously, namely: 1) sustainably increasing agricultural productivity and incomes, 2) building resilience to the impacts of climate change by implementing biopesticide regimes and reducing conventional chemical pesticide use, and 3) contributing to climate change mitigation, where possible ( FAO 2017).

The rising consumer demand for sustainably produced food, coupled with the growing resistance of pests to conventional chemical pesticides, the emergence of novel pest threats, and the impacts of climate change, underscores the increasing importance of biopesticides in the future of climate-smart agriculture systems ( Chandler et al. 2008). To address this pressing issue, adopting environmentally safe and host-specific pest control options alongside IPM programs can create a winning combination for growers ( Chang et al. 2003; Essiedu et al. 2020).

Microbial biopesticides are a broad range of pest control products obtained from microorganisms, including bacteria, viruses, fungi, protozoa, and entomopathogenic nematodes. Many microbial biopesticides can be cost-competitive with other pesticides and fungicides ( Ruiu 2018). Microbial biopesticides are the most commonly used in pest control programs ( Kvakkestad et al. 2020). Most entomopathogenic bacteria belong to the *Bacillaceae* family, but the most widely used commercial bacterial biopesticides are from the genus *Bacillus*, especially the species *Bacillus thuringiensis* ( *Bt*) and *Bacillus subtilis* ( *Bs*) ( Alsaedi et al. 2017; Akutse et al. 2020). These are aerobic bacteria that produce insecticidal crystal proteins that are toxic to specific insect orders ( Gill and Cowles 1992; Raymond et al. 2009). Over 200 *Bt*-based biopesticides have been registered ( Desneux et al. 2022) and are effective against various lepidopterans, coleopterans, dipterans, and hemipterans. However, resistance to some *Bt* products has been noted. For instance, laboratory resistance to *Bt kurstaki* has been reported for diamondback moth larvae ( Liao et al. 2019), tobacco budworm, and beet armyworm ( Blanco et al. 2009; Muhammad et al. 2019). More recently, western corn rootworms have shown field resistance to *Bt* ( Legwaila et al. 2015). *Actinomycetes* - filamentous, aerobic bacteria resembling fungi - are naturally found in soils and have also been developed into biopesticides ( Pitterna et al. 2009). Some *Actinomycete* species can kill insects, including *Tuta absoluta.* Two species, *Saccharopolyspora spinosa* and *Streptomyces avermitilis*, are used to make bacterial biopesticides ( Akutse et al. 2020). *Spinosad*, a microbial biopesticide made from the aerobic fermentation of *S. spinosa* contains secondary metabolites called *spinosyns* ( Higa 1994).

Most entomopathogenic fungi belong to the two orders, *Entomophthorales* and *Hypocreales* (previously *Hypomycetes*) ( Hajek and Leger 1994). The majority *of entomophthoralean* fungi are obligate parasites with limited host ranges and generate both sexual and asexual spores. Using epizootics, many of these species inhibit insects, the most prevalent genera being *Entomophaga, Entomophthora,* and *Zoophthora* ( Goettel and Inglis 2000). *Beauveria spp., Metarhizium, Isaria,* and *Lecanicillium* spp. are fungi that infect and kill insect pests through direct contact or by penetrating their cuticle, following which the spores germinate and multiply in the insect’s body ( de Faria and Wraight 2007; Kumari et al. 2022). Viruses from families of baculoviruses, cypoviruses, and densoviruses have been successfully used as viral biopesticides ( Abd-Alla et al. 2020). For example, the *nucleopolyhedro* virus effectively infects and kills insect larvae ( Cory 2000) and is used to control a variety of serious insect pests, especially moths ( Gramkow et al. 2010). Insect viruses are considered environmentally safe due to their high selectivity and lack of non-target organisms species shifts. They also degrade naturally and do not persist in the environment ( Abd-Alla et al. 2020). Despite their advantages, insect viruses face certain challenges as microbial biopesticide agents. These include their slow action, narrow host range, and potential for resistance development in insect hosts ( Abd-Alla et al. 2020). Addressing these challenges through research and development efforts is crucial to fully realize the potential of insect viruses as sustainable pest control solutions.

**Figure 1.**
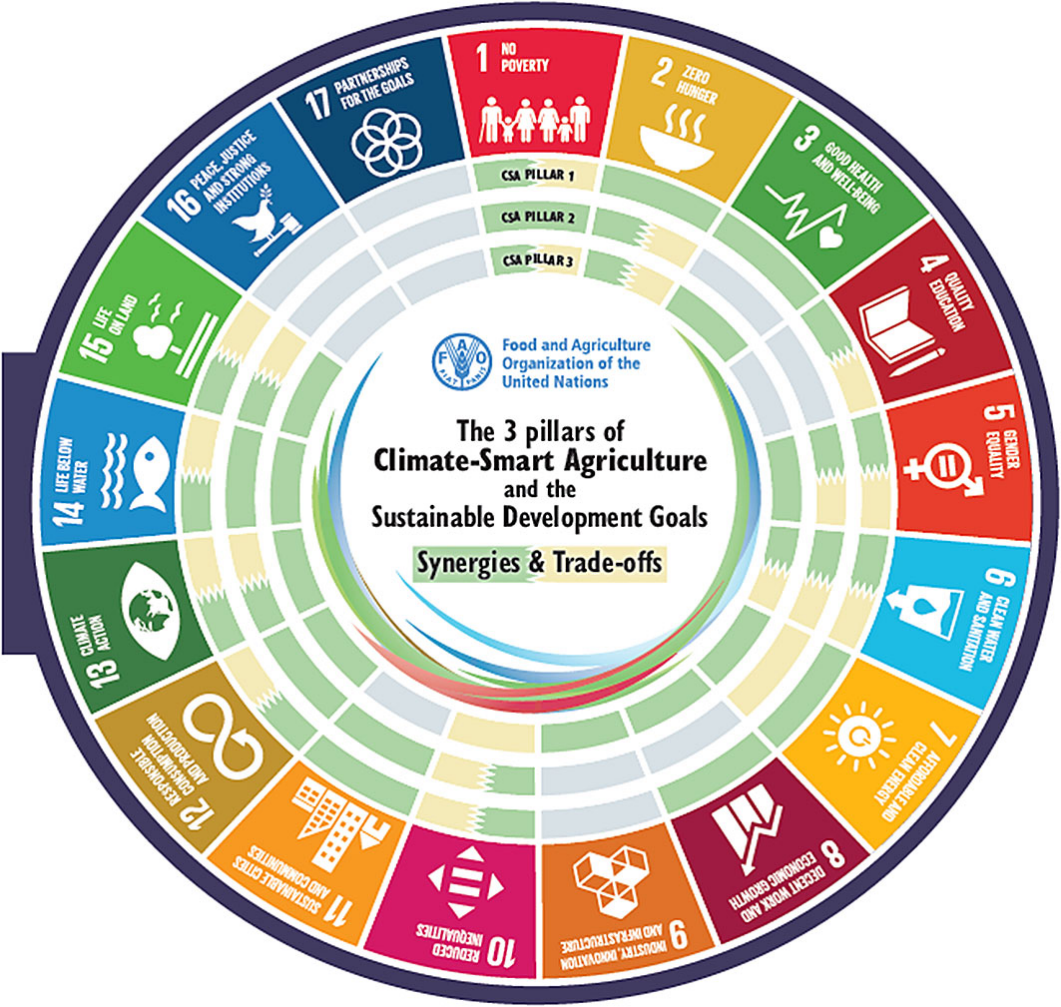
The 3 pillars of Climate-Smart Agriculture and the Sustainable Development Goals ( FAO, 2019). The visual mapping illustrates how the three pillars of Climate smart agriculture (CSA) interact with and impact each of the 17 Sustainable Development Goals (SDGs). The second part demonstrates how the five steps for implementing CSA intersect with and affect the 17 SDGs in terms of synergies and trade-offs.

Generally, microbial biopesticides can have shorter shelf lives compared to chemical pesticides. They may also be sensitive to environmental factors such as sunlight, temperature, and moisture ( McManus 1989; Matos et al. 2020; Wattimena and Latumahina 2021). However, if stored according to the label instructions, they could last up to 3-6 months ( Akutse et al. 2020). There is a potential for microbial biopesticides to be integrated into IPM strategies in agriculture without significantly compromising productivity and yield ( Aroraet al. 2020). However, several limitations currently hinder the widespread adoption of microbial biopesticides. These include 1) the high cost of refined commercial products, 2) policy and regulatory issues, 3) challenges in meeting global market demand, 4) lack of standardized preparation methods and guidelines, 5) difficulties in determining appropriate dosages of active ingredients, 6) susceptibility to environmental factors, 7) limited stability, and 8) slow adoption by end-users. While research breakthroughs are expected to address these limitations in the coming years, farmers, particularly those in rural areas, can still benefit from using microbial solutions as a means of plant protection to enhance crop quality and ensure farms are productive and profitable ( Stevenson et al. 2017). It is important to note that the maximum effectiveness of biopesticide applications, including microbial biopesticides, is achieved when they are integrated into an overall IPM approach ( Quarles 2013) and aligned to the 3 pillars of climate-smart agriculture ( FAO 2017) ( Figure 1). Climate-smart agriculture (CSA) has a strong potential to contribute to achieving the 17 Sustainable Development Goals (SDGs). A critical precondition for realising this scope is identifying potential synergies and trade-offs between the three pillars of CSA and the five implementation steps of CSA and the SDGs. These provide entry points for targeted CSA planning to enhance synergies and reduce trade-offs. CSA and the SDGs have synergies and trade-offs at different levels for each of the three pillars of CSA, where some of the synergies & trade-offs overlap all three pillars of CSA for some of the SDGs, namely SDGs 2 and 6. The overlapping synergies & trade-offs for the implementation steps and SDGs are different, as only step 2 has overlapping trade-offs and synergies in SDGs 6, 6, 9, 13, and 15 ( United Nations 2015).

## 2. Methods

To comprehensively gather information on biopesticides, we employed a systematic and rigorous approach. We utilized a wide range of electronic sources, including Google Scholar, PubMed, Scopus (Elsevier), Web of Science, Semantic Scholar, Academia, and other relevant websites, to conduct extensive literature searches. Our analysis of over 200 scientific papers and other relevant online resources enabled us to amass a comprehensive archive of pertinent literature. Only microbial (bacterial, fungal, viral, nematode, and protozoa) biopesticides and other types were excluded. Our primary focus was on recent advancements in the development and application of biopesticides within the framework of IPM within the climate-smart agriculture framework. The literature outcomes were categorized into the (i) classification of biopesticides, (ii) importance of biopesticides in sustainable agriculture, (iii) role of microbial biopesticides in integrated pest management, (iv) different types and formulations of microbial biopesticides, and (v) advancement and future perspectives of microbial biopesticides.

## 3. Concept and classification of biopesticides

Biopesticides are defined by the US Environmental Protection Agency (USEPA) as ‘naturally occurring substances, microorganisms, and plant-produced substances that control pests’( EPA 2023). While the USEPA defines biopesticides as “pesticides derived from naturally occurring substances”, this term is not universally recognized. According to the Southern African Development Community ( SADC), the term biopesticide is generally applied to a substance derived from nature, such as a microorganism or botanical or semiochemical, that may be formulated and applied like a conventional chemical pesticide and that is normally used for short-term pest control ( SADC 2019). The East African Community ( EAC) also classified the term “biopesticides” as microbial, macrobial, botanical, and semiochemical biopesticides derived from or based on genetically modified organisms (GMOs) and pest control agents. The International Biocontrol Manufacturer’s Association (IBMA) and the International Organization for Biological Control (IOBC) prefer the term “biocontrol agents” (BCAs) instead. IBMA classifies BCAs into four groups, namely macrobiotics (predators and parasites), microbial (bacteria, fungi, and viruses that kill or harm pests), natural products (plant extracts), and semiochemicals (chemical signals) ( Guillon 2003). The European Environmental Agency define biopesticide as “a pesticide in which the active ingredient is a virus, fungus, bacteria, or a natural product derived from a plant source” ( European Environment Agency 2023).

Biopesticides offer several advantages over conventional chemical pesticides, including lower toxicity to non-target organisms, reduced environmental impacts, and less likelihood of developing resistance to pests. Biopesticides effectively control insects, diseases, weeds, and nematodes, promote plant health, and enhance the productivity and profitability of farmers. Importantly, many of them are non-toxic to beneficial organisms and wildlife. Additionally, their biodegradability reduces pollution concerns associated with many chemical pesticides ( Shilpi and Promila 2012; Essiedu et al. 2022). Due to their targeted action and effectiveness in small quantities, biopesticides are gaining wide application on plants and crops. They are playing an increasingly significant role in agriculture, promoting sustainable practices and a healthier environment ( EPA 2023), thus complying with the climate-smart agriculture principles ( FAO 2017), and, ultimately, the Paris Agreement ( United Nations 2015), the Nagoya Protocol of Article 6 (Access to Genetic Resources) and Article 7 (safeguards the rights of indigenous and local communities regarding their traditional knowledge and ensures they benefit from its use) ( The Nagoya Protocol 2014).

### 3.1 Mechanism of action and efficacy of biopesticides

*How do they work?* Biopesticides work through various mechanisms, such as 1) biochemical disruption,-where they interfere with the biochemical processes of pests, affecting their growth, development, or reproduction; 2) microbial activity-infecting or parasitizing pests; 3) predation and competition-acting as natural predators or competitors of pests to limit their population growth; and 4) repellency-deterring pests from attacking crops ( Essiedu et al. 2022). They can play a crucial role in IPM strategies and contribute to sustainable agricultural practices by reducing the environmental impact and minimizing the risk of pesticide resistance ( Essiedu et al. 2022), thereby complying with the 3 pillars of climate-smart agriculture and the synergies with the SDGs ( FAO 2024) ( Figure 1).

## 4. Importance of biopesticides in sustainable agriculture

Biopesticides are an important tool in sustainable agriculture, and their use will likely continue growing. The use of biopesticides has steadily increased at a Compound Annual Growth Rate ( CAGR) of 11.0% between 2018 and 2022 ( CAGR) between 2018 and 2022. The global biopesticides market reached a value of US$ 8,123.8 million in 2023. This upward trend is expected to continue, with the market expanding at a 10.3% CAGR and reaching US$ 21,827.6 million by 2033 ( Persistence Market Research 2023). Food security is essential due to the world’s growing population. Humans and global economies depend on a stable and reliable food supply. As the population grows, food security will become more threatened ( FAO 2024). Sustainable farming and maximizing soil fertility require non-toxic and environmentally sustainable pesticides. Thus, the need for biopesticides will likely increase over time ( Bharti and Ibrahim 2020). Sustainable farming depends on biopesticides, which are poised to advance significantly in the coming years ( Copping et al. 2000; Alavanja 2009). Optimizing manufacturing processes to increase yields and reduce costs can make biopesticides more economically viable. By using biopesticides, farmers can reduce their reliance on chemical pesticides, protect human health and the environment, and produce safe and healthy food ( Hezakiel et al. 2023; FAO 2024).

## 5. Role of biopesticides in integrated pest management

Biopesticides play a vital role in IPM programs, which aim to control pests effectively whilst reducing the reliance on conventional chemical pesticides ( Jozsef 2020). As the demand for more sustainable, environmentally safe, and sustainable agricultural practices grows, the use of biopesticides is becoming increasingly popular as part of IPM strategies. IPM aims to combine multiple pest control methods, including biopesticides, to minimize the use of chemical pesticides and maintain a balanced ecosystem in agricultural environments ( Jozsef 2020). When used within IPM programs, biopesticides offer several advantages, including 1) targeted pest control, 2) diverse modes of action, 3) resistance management, 4) reduced negative environmental impact ( Isman 2006), 5) sustainable crop production ( Gurr et al. 2017), and 6) reduced risk of pesticide resistance in non-target organisms ( Prasanna et al. 2018). Many biopesticides have the potential to contribute to economic growth and rural livelihood improvement, thus complying with the SDGs ( Figure 1).

Additionally, biopesticides offer potential benefits in the face of climate change challenges, and they align with the growing consumer demand for environmentally friendly and sustainable agricultural practices ( FAO 2017; Akutse et al. 2020). Besides, biopesticides 1) generate fewer greenhouse gasses (GHG) than chemical pesticides, 2) improve nutrient availability for plants, 3) increase soil fertility, and 4) offer a more environmentally safe approach to pest control in agriculture, which can be useful in the fight against climate change.

### 5.1 Biopesticide categories

Biopesticide is a generic term generally applied to a substance derived from nature, such as a botanical or semiochemical, that may be formulated and applied like a conventional chemical pesticide and that is normally used for short-term pest control ( USEPA 2020). Naturally derived or laboratory-made chemicals with structures and functions similar to naturally occurring ones are known as biochemical pesticides. They differ from traditional pesticides in their origin (source) and how they control or kill pests ( O’Brien and Jones 2009). Microbial pesticides are natural pest control agents harnessing the power of microscopic living organisms ( Clemson 2007). These “living bullets” can be either spores or active organisms, often specifically chosen for their pathogenic nature towards targeted pests. Common examples include biofungicides ( *Trichoderma, Pseudomonas, Bacillus, Colletotrichum*), bioherbicides ( *Phytophthora, Cylindrobasidium, Colletotrichum acutatum*), and bioinsecticides ( *Bt*) ( MacGregor 2006; Gupta 2010). Microbial biopesticides can originate from naturally occurring or genetically modified bacteria, fungi, algae, viruses, or protozoans. They subdue pests through a diverse range of strategies (toxic metabolites, disease-causing, competitive exclusion, and other modes of action), depending on the specific microbe ( Clemson 2007).

Microbial biopesticides offer diverse delivery methods to effectively reach crop targets. These methods include live organisms, dead organisms, and spores ( O’Brien and Jones 2009). Globally, microbials account for 41% of all BCAs used worldwide, macrobiotics comprise 33% of the global market, and other natural products represent the remaining 26% ( Guillon 2003). Based on their origin or active ingredients, they can be classified as bacterial-based biopesticides, fungal biopesticides, viral biopesticides, nematodes, and protozoan-based biopesticides.

### 5.2 Bacterial-based biopesticides

**Figure 2.**
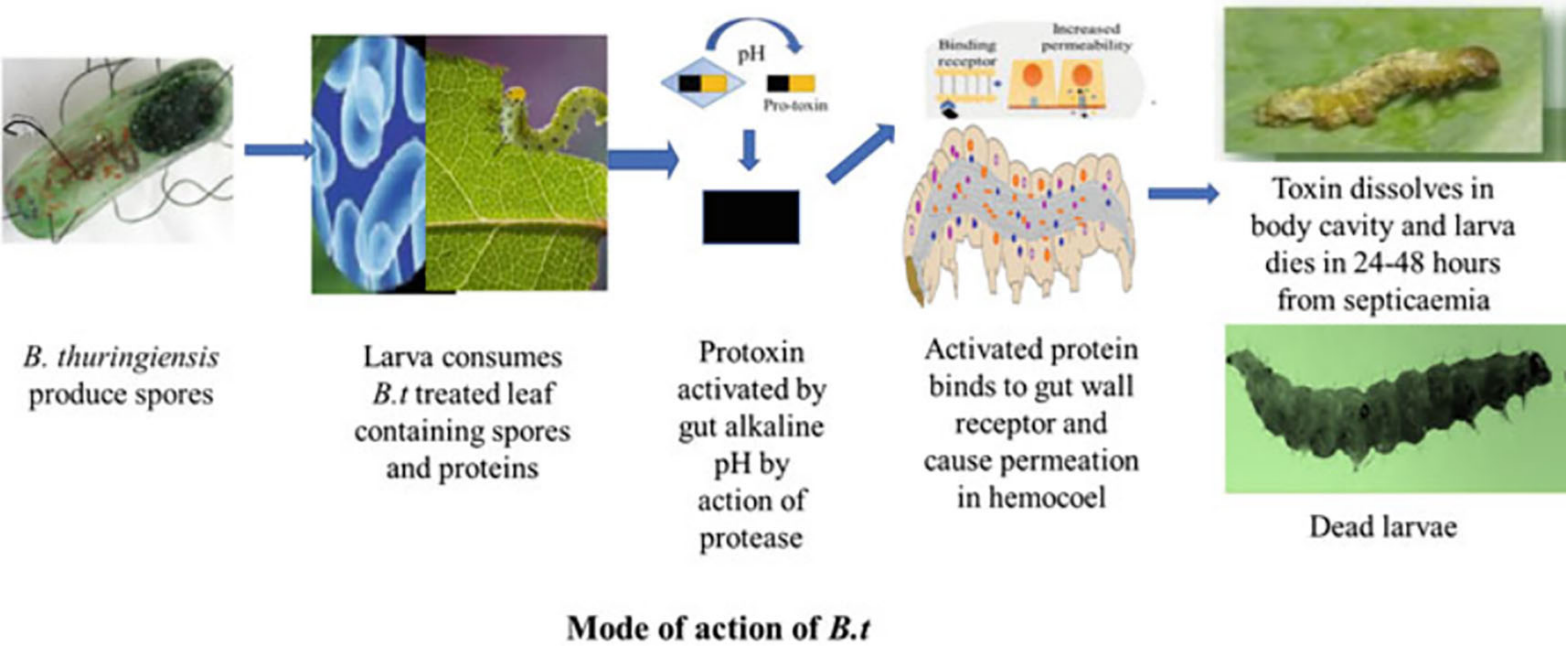
Effects of *Bacillus thuringiensis* (Bt gene and Cry protein) on insect larvae ( Singh et al. 2019).

Among all microbial pesticides, bacterial biopesticides are the most widely used. These versatile microbes attack pests in multiple ways, primarily targeting insects. While mostly used against various orders of insect pests in agriculture, bacterial biopesticides can also curb the growth of plant-harming bacteria and fungi ( O’Brien and Jones 2009; Jouzani and Valijanian 2017). Bacterial pathogens need direct contact with their target to be effective, often requiring ingestion. They secrete endotoxins, protein-based toxins specific to the targeted pest that wreak havoc on their digestive system ( Schallmey and Singh 2004). *Bacillus thuringiensis (Bt)* dominates the biopesticide market, accounting for a whopping 90% share in the USA alone ( Chattopadhyay and Bhatnagar 2004). *Bacillus thuringiensis* is the most common species used commercially to control a diverse range of insect pest species, including lepidopterans, hemipterans, and coleopterans in agriculture, forestry, and even medicine since its discovery in 1901 ( Mazid 2011). It is a spore-forming bacterium with several strains that produce diverse insecticidal crystal proteins, often targeting different pests ( Table 1). The crystal proteins are toxic and, once ingested, damage the gut tissues, leading to paralysis of the gut. The infected insects stop feeding and eventually die from gut impairment and starvation. *Bt* var. *kurstaki* specifically targets caterpillars, while the genes encoding Cry proteins have been transferred into crops like cotton, hence reducing reliance on chemical pesticides ( Mazid 2011). Commercial *Bt*-based products are available in various forms, such as powders containing a combination of dried spore and crystal toxins or formulations in liquid suspensions ( Boyetchko et al. 2020). *Bacillus thuringiensis’s* high target specificity and environmental safety make it an ideal complement to traditional chemical pesticides for insect pest control ( Roy and Moktan 2007; Kumar 2012). Figure 2 illustrates the effects of *Bacillus thuringiensis* on insect larvae. Other entomopathogenic bacteria of interest include *Chromobacterium subtsugae, Brevibacillus laterosporus, Lysinibacillus sphaericus, Paenibacillus popilliae, Serratia marcescens*, and *Yersinia entomophagy* ( Gangwar et al. 2021).

**Table 1.** Microbial biopesticide categories, mode of action, and their use in pest management.

Fungal-based biopesticides	Application type and mode of action	Use	Remark
*Beauveria bassiana*	It infects the host insect through the cuticle, colonizes its body, and ultimately causes death ( Baldiviezo et al. 2020). This fungus is known for its rapid multiplication and the production of various toxins that result in exogenous infections ( Nakahara et al. 2009; Naqqash et al. 2016).	Widely used fungal biopesticides are effective against numerous insect pests ( Naqqash et al. 2016).	It is among the most effective biocontrol fungi ( Naqqash et al. 2016).
*Metarhizium anisopliae*	It can infect and kill a variety of insect pests, and this product is suitable for use in non-food areas such as ornamental greenhouses, nurseries, residential and institutional lawns, and landscape perimeters. However, it should not be used in areas where there is a risk of water contamination ( Naqqash et al. 2016; Montalva et al. 2016). It can also be used for the control of mosquitoes in the public health domain ( Vivekanandhan et al. 2022)	This product targets a variety of pests, including ticks and beetles, as well as aphids, mealybugs, fruit flies, root weevils, grasshoppers, whiteflies, gnats, and thrips. etc. ( Montalva et al. 2016), and mosquitos ( Vivekanandhan et al. 2022). *M rileyi* controls lepidopterans.	It can be applied at terrestrial non-food sites and as indoor residual spraying for mosquitoes.
*Verticillium lecanii* also known as *Lecanicillium lecanii*	This product includes fragments of *Verticillium lecanii* fungi, along with spores. When these spores encounter the outer layer of a targeted pest insect, they germinate, penetrate their cuticle, and proliferate within their bodies. This eventually leads to the insect’s demise, as it becomes drained of nutrients ( Lee et al. 2006).	Used for controlling aphids, whiteflies, and other sucking insects from fruits, vegetable crops, etc. ( Lee et al. 2006; Feng et al. 2000). *Lecanicillium lecanii* targets whiteflies, leafminers, aphids, and scale insects ( Ruiu 2018).	This agro-input is harmless, environmentally safe, and cost-effective.
*Paecilomyces lilacinus*		Controls plant parasitic nematodes ( Akutse et al. 2020).	
*Conidiobolus thromboides* Acari	Hemiptera, Thysanoptera		
*Isaria fumosorosea*		*I. fumosorosea* targets soft-bodied insects such as whiteflies ( Akutse et al. 2020).	
*Trichoderma* sp.	Trichoderma spp. is a type of fungicide that is highly effective in combating soil-borne diseases like root rot. It is especially beneficial for dryland crops such as groundnut, black gram, green gram, and chickpea, which are prone to these diseases ( Ghayur 2000).	They are known for their antagonistic interactions with various plant pathogens, including fungi, bacteria, and nematodes ( Ghayur 2000). *Trichoderma asperellum* against soil-borne pests.	The use of *Trichoderma* spp. As a biopesticide is promising to control plant diseases and insect pests. ( Howell 2003).
*Phytophthora palmwora MWV*	Liquid bioherbicide formulation	used to control stranglervinrie weed.	( Kachhawa 2017)
*Colletotrichum gIloeosponoides*	A dry powder bioherbicide	Targeted to control Northern joint vetch	( Kachhawa 2017)
*Colletotrichum acutatum*	A liquid bioherbicide formulation	Targeted to control *Hakea sericea* and *H. gibbosa*	it is used in agriculture, forestry, and conservation applications to control these alien and invasive species ( Muir 2023).
**Bacterial-based pesticide**			
*Bacillus thuringiensis (Bt)*	The most widely used biopesticide worldwide is Bt. It primarily targets lepidopterous pests, which are known to cause grave damage. Some examples are American bollworms in cotton and stem borers in rice. It releases toxins that harm the midgut of the pest when the larvae consume it, leading to its demise ( Ghayur 2000).	Lepidopteran pests, such as stem borers in rice.	It is a highly specific, safe, and effective organism for insect control ( Roh et al. 2007).
*B. subtilis*	Produce a variety of antimicrobial compounds, such as antibiotics, lipopeptides (e.g., surfactin, iturin), and cyclic peptides (e.g., bacillomycin), which inhibit the growth and activity of plant pathogens( Djaenuddin et al. 2020).	This product targets pests such as wilts, crown rot, root rot, and other seed-borne diseases that are caused by fungi such as *Fusarium, Aspergillus, Pythium, and Rhizoctonia* ( Djaenuddin et al. 2020).	It is safe for the environment and humans.
*Btvar kurstakz*	This product is used as a bioinsecticide	It is used to target *Lepidopteran* spp.	No side effects to non-target organisms.
*Bt israelensis*	controls gnats fly	It is used to control gnats flies	( Ruiu 2018)
*Bt tenebrionsis*	*Beauveria bassiana*	shows activity against Coleopteran adults and larvae	( Rajaput et al. 2019)
*Bt. Vnar. sun dlego* *Bt var. tenebnoms*	Both are bioinsecticides effective against targeted pests.	Developed by Ecogen and Thermo Trilogy company to control Coleopteran pests.	No phytotoxicity effects
*Pseudomonas fluorescens-*	*Pseudomonas* species are significant bacteria in agriculture. Studies have demonstrated that they can enhance plant growth and shield them from pathogens and herbivores ( Daniel 2020).	It can colonize the rhizosphere and produce secondary metabolites that suppress soil-borne pathogens ( Glick 2012). These bacteria also play a role in phytoremediation and are part of the essential microbiome of numerous plants ( Adesemoye and Kloepper 2009; Germaine et al. 2009).	No toxicity effects to non-target organisms.
*Streptomyces* species	Filamentous bacteria that produce various antifungal compounds. *Streptomyces strains* possess valuable applications in agriculture due to their ability to biologically control phytopathogens, particularly fungi that are harmful to plants ( Law et al. 2017).	They have been utilized as biocontrol agents against plant pathogenic fungi, such as *Fusarium* and *Phytophthora* species ( Gonzalez-Franco 2009; Ara et al. 2014).	Streptomyces strains display antibacterial, antifungal ( Ara et al. 2014), and immunosuppressive tendencies *.*
**Viral-based biopesticides**			
*Cydia pomonella* granulovirus *-* (CpGV)	They are commonly used for the control of the codling moth ( *Cydia pomonella*), a major pest of apple and pear orchards. *CpGV* is a safe, effective, and environmentally friendly biocontrol agent for codling moth ( EPPO 2019).	The CpGV virus particles infect and kill the codling moths’ larvae, reducing pest populations ( EPPO 2019).	It is a valuable tool for sustainable agriculture.
Baculoviruses	Viruses are aimed at specific targets and are capable of infecting and eliminating various significant plant pests. Known as baculovirus insecticides. Nucleopolyhedroviruses (NPVs) control *Helicoverpa armigera, Helicoverpa spp. Spodoptera exigua, S litura.* Granuloviruses (GVs) control *Plutella xylostella, Thaumatotibia leucotreta*, *Pieris rapae, Cydia pomonella*	These viruses have been proven to be highly effective against pests such as lepidopterous in cotton, rice, and vegetables ( Chang et al. 2003; Ruiu 2018; Kumar et al. 2021).	They are insect-specific viruses widely used as biopesticides.
*Cylindrobasidium laeve*	A liquid bioherbicide formulation	Targeted for the control of *Acacia mearnsii* and *A. decurrans*	it is used in agriculture, forestry, and conservation applications to control these alien and invasive species ( Muir 2023).
**Nematode based biopesticides**			
*Steinernema* spp. and *Heterorhabditis* spp.		are used on different host crops to control pests such as leafminers, thrips, weevils, beetles, cutworms, scarab grubs, lepidopterans, and fungus gnats.	( Koppenhöfer et al. 2020; Tarasco et al. 2023; Rae et al. 2023).
*Phasmarhabditis hermaphrodita*		used against several slug and snail species.	( Rae et al. 2023)
*H. marelatus*		It is used to control white grubs (scarabs), cutworms, and black vine weevils.	
*H. bacteriophora*	White grubs (scarabs), cutworms, black vine weevils, flea beetles, corn rootworms, citrus root weevils ( *Diaprepes* spp.) ( Kachhawa 2017)		
*H. megidis*	Used to control weevils		
*S. glaseri*	Used to control white grubs (scarabs, especially Japanese beetle, *Popillia* sp.), banana root borer ( Kachhawa 2017)		

Recombinant DNA technology has been used to develop several new Bt-based products. MVP ^TM^ and M-Trackr ^TM^, for example, were created using Mycogen Corporation’s CellCap ^®^ encapsulation method. This procedure entails removing a gene from *Bt* that encodes the delta-endotoxin protein, incorporating it onto a plasmid, and inserting it into a *Pseudomonas fluorescens* strain ( Pitterna et al. 2009; Legwaila et al. 2015). Before being killed by heat and chemical treatment, the recombinant cells were cultured in aerobic culture and induced to express delta-endotoxin. The dead bacterial cells in the aqueous formulation acted as microcapsules, protecting the fragile *Bt* toxin from destruction in the environment.

Pathogenic bacteria have also been considered for biological weed control. However, one of the challenges to using phytopathogenic bacteria for biological weed control is the necessity of free water for dispersal and wounds or natural holes for bacterial penetration into the weed plant ( Zidack et al. 1992; Johnson et al. 1996). Previously, researchers have studied *Xanthomonas campestns pv. Poae* for the control of annual bluegrass ( *Pea annua* L.) and discovered that cutting or mowing turfgrass allows bacteria to enter the plant ( Imaizumi et al. 1997). Furthermore, bacteria administered at a rate of log CFU/mL at high water levels (400 mL/m ^2^) reduced disease severity in annual bluegrass by more than 90%. Silwet L-77 (0.2%), an organosilicon surfactant, increased bacterial penetration and entrance into plant stomata and hydathodes ( Johnson et al.1996; Boyetchko et al. 2020). A low surface tension of 30 dynes/cm or less is necessary to convey liquid into a leaf’s stomata. Silwet decreases the surface tension of water to 20 dynes/cm. Compared to plants sprayed with bacteria without the surfactant, the application of *Pseudomonas syringae* pv. *Tagetes* with this surfactant facilitated the penetration and entry of bacteria into stomata and hydathodes, significantly increasing disease severity and incidence ( Johnson et al. 1996).

Bacterial biopesticides are environmentally safe and precise alternatives to traditional pest control methods. They offer several advantages, including safety for humans, wildlife, and beneficial insects. These biopesticides can be used in conjunction with other pest control strategies, including some chemical methods. Additionally, they leave no harmful residues, don’t harm pollinators and other valuable organisms, and can be applied close to harvest time. The additional benefit is that they can self-propagate, extending their effectiveness beyond the initial application and into subsequent growing seasons ( Meenatchi and Aditi 2021). Bacterial biopesticides, while effective, come with a few disadvantages. For instance, they only target specific species or groups of insects, leaving other pests to survive and continue causing damage. This means that additional biopesticides are needed to complement the treatment when dealing with different pests. Hence, they should be applied alongside other IPM strategies. Finally, the effectiveness of microbial insecticides can be influenced by factors like ultraviolet radiation and heat, so it’s important to apply them strictly according to the label ( Meenatchi and Aditi 2021).

### 5.3 Fungal biopesticides

Mycopesticides include naturally occurring fungi and fungi cell components. These fungi attach to their targets through sticky spores. These spores then germinate and form microscopic tubes that pierce the insect’s skin, releasing potent hydrolytic enzyme mixtures along with toxins ( Langner and Göhre 2016). This internal invasion disrupts the insect’s physiology, leading to its eventual death. The fungi then grow outward from the dead insect, producing new spores to continue the cycle, hence perpetuating natural pest control ( Pineda et al. 2007; Gabarty et al. 2014). The role of hydrolytic enzymes, especially chitinases, in the killing process, and the possible use of chitin synthesis inhibitors are prime research areas. Popular commercially available mycoinsecticides are often derived from species like *Beauveria bassiana, Metarhizium anisopliae, Isaria fumosorosea, Hirsutella thompsonii, Phytophthora palmivora, Alternaria cassia*, and *Lecanicillium spp.* ( Table 1). Notably, *Beauveria bassiana and Metarhizium anisopliae* are two common ascomycetes known for their broad effectiveness against pests like aphids, beetles, grasshoppers, and caterpillars. These fungi are typically applied as conidia (spores) or mycelium, which then sporulate after application, ensuring sustained pest control ( Lacey et al. 2015). *Cylindrobasilium laeve* for the control of *Acacia mearnsii* (black wattle) and *Colletotrichum acutatum* for the control of *Hakea sericea* (silky hakea) are some of the available mycoherbicides for integrated alien and invasive species management.

Fungal biopesticides hold immense promise as environmentally safe options against a diverse range of insect and mite pests. They possess several desirable traits for biocontrol: they selectively target pests, leaving beneficial insects like bees and predators unharmed. Additionally, they generally pose no threat to the growth or development of other beneficial organisms like earthworms and springtails. This makes them ideal candidates for IPM and sustainable agricultural practices, promoting biodiversity and protecting the environment ( Goettel et al. 2008; Kim and Goettel 2011; Koike et al. 2011). Several fungi have been successfully mass-produced and formulated for commercial application as mycoinsecticides, paving the way for their widespread use in pest control ( Chandler et al. 2008).

The remarkable potential of entomopathogenic fungi is actively being harnessed for use in IPM programs. Mass production techniques and ecological approaches are constantly refined to optimize their effectiveness and ensure seamless integration into sustainable agricultural practices ( Chandler et al. 2011; Lacey et al. 2015). Furthermore, promising results have been achieved by combining fungal biopesticides with traditional insecticides, often leading to significantly boosted pest mortality rates. For example, studies have shown that combining *B. bassiana* with low-dose insecticides enhances potato beetle control, while its synergy with neem oil proves effective against tobacco thrips eggs and nymphs ( Chandler et al. 2011; Lacey et al. 2015; Sarwar 2015).

Fungi-based biopesticides boast several advantages over other biocontrol methods. Their broader host range allows them to tackle a diverse range of pests across fields, greenhouses, storage facilities, and soil, unlike bacteria and viruses with limited targets ( Amruta et al. 2019). Commercially important fungi like *Beauveria, Metarhizium, Lecanicillium,* and *Isaria* are surprisingly easy to mass produce, requiring significantly less substrate compared to alternative methods. These fungi are highly productive, delivering powerful, targeted pest control while reducing the impact on the environment. They pose no risk to beneficial organisms, readily biodegrade, and integrate seamlessly into IPM programs. Additionally, their persistence in the environment offers long-lasting protection against pest outbreaks, making them a potentially cost-effective solution for sustainable agriculture ( Naveenkumar et al. 2017; Rajaput et al. 2019; Sufyan et al. 2020; Upamanya and Bhattacharyya 2020).

**Figure 3.**
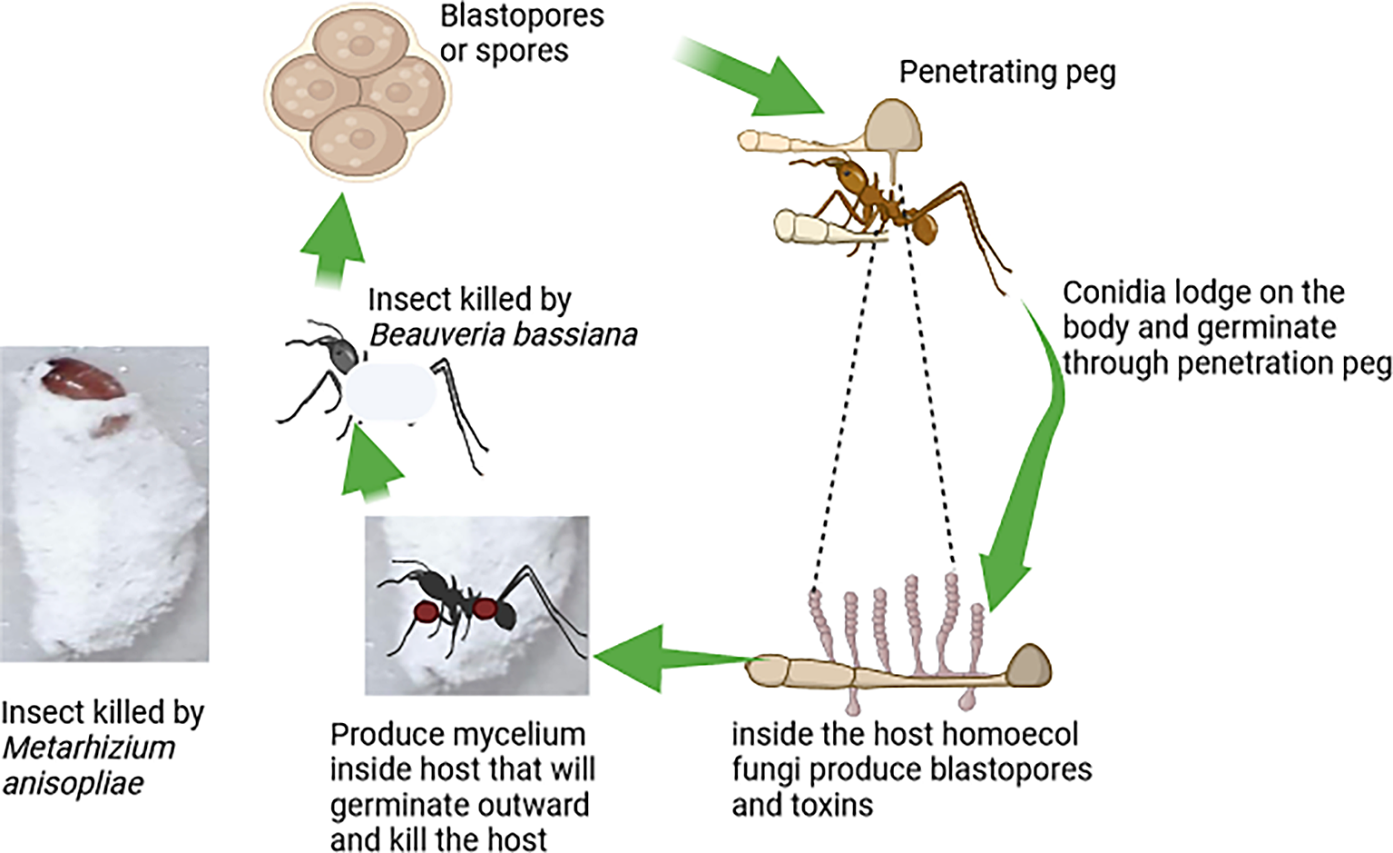
Mode of action of fungi-based biopesticides.

Fungi-based biopesticides, however, face some environmental hurdles. Optimal spore germination and penetration into insect cuticles often require humidity levels exceeding 80%, depending on the specific fungal species. Temperature fluctuations and exposure to UV radiation can significantly impact their survival. Germinating conidia, essential for active pest control, are often delicate and susceptible to environmental damage. Conversely, the production of durable resting spores for long-term persistence can be expensive compared to other microbial methods like bacteria-based biopesticides ( Sabaratnam 2002; Kolombet et al. 2008; Kordali et al. 2008; Li et al. 2014). Figure 3 illustrates the mode of action of fungi-based biopesticides, highlighting the steps by which these biopesticides attack target insect pests ( Figure 3).

### 5.4 Viral biopesticides

Baculoviruses (BVs) are tiny, naturally occurring viruses that have emerged as powerful allies in the fight against insect pests. They belong to a group known as entomopathogenic viruses, specifically targeting and infecting insects and other arthropods. Unlike harmful conventional chemical pesticides, baculoviruses pose no threat to humans, wildlife, or beneficial insects, making them an environmentally safe option for pest control ( Cory 2000; Jackson and Crawford 2005; Ramle et al. 2005; Moscardi et al. 2011), and are considered as the most commercial viral biopesticides ( Granados 1986; Moore and King 1987). Baculoviruses involve circular supercoiled double-stranded DNA genomes, which range from 80 to 180 kbp ( Rohrmann 2013). Within the *Baculoviridae* family, scientists have identified three main subgroups: nuclear polyhedrosis viruses (NPVs), cytoplasmic polyhedrosis viruses (CPVs), and granulosis viruses (GVs). These subgroups differ in the structure and number of their protective protein coats, called occlusion bodies ( Moscardi et al. 2011). These bodies allow the virus to survive outside the host, waiting for its next target. NPVs and CPVs form polyhedral bodies with numerous virus particles, while GVs have smaller, granular bodies containing just one particle. Notably, each subgroup employs a unique method to uncoat and establish infection within their host: NPVs in the nucleus, CPVs in the cytoplasm, and GVs within the nuclear pore complex ( Cory 2000; Moscardi et al. 2011).

*Baculoviruses* offer highly specific targeting, primarily focused on lepidopteran larvae and hymenopterans (butterfly and moth) pests that damage crops like cotton, rice, and vegetables. One example is *Heliothis zea nucleopolyhedrosis* virus (HzNPV), the first commercially successful broad-spectrum viral insecticide. Interestingly, HzNPV demonstrates versatility, effectively controlling pests across various crops like soybeans, sorghum, maize, tomatoes, and beans. While other entomopathogenic viruses exist, such as tetraviruses and cypoviruses, their use in crop protection remains limited compared to the widespread adoption of baculoviruses ( Sarwar 2015). A comprehensive summary of viral biopesticides and their target pests is presented by ( Usta 2013; Singh et al. 2019; Meenatchi and Aditi 2021).

**Figure 4.**
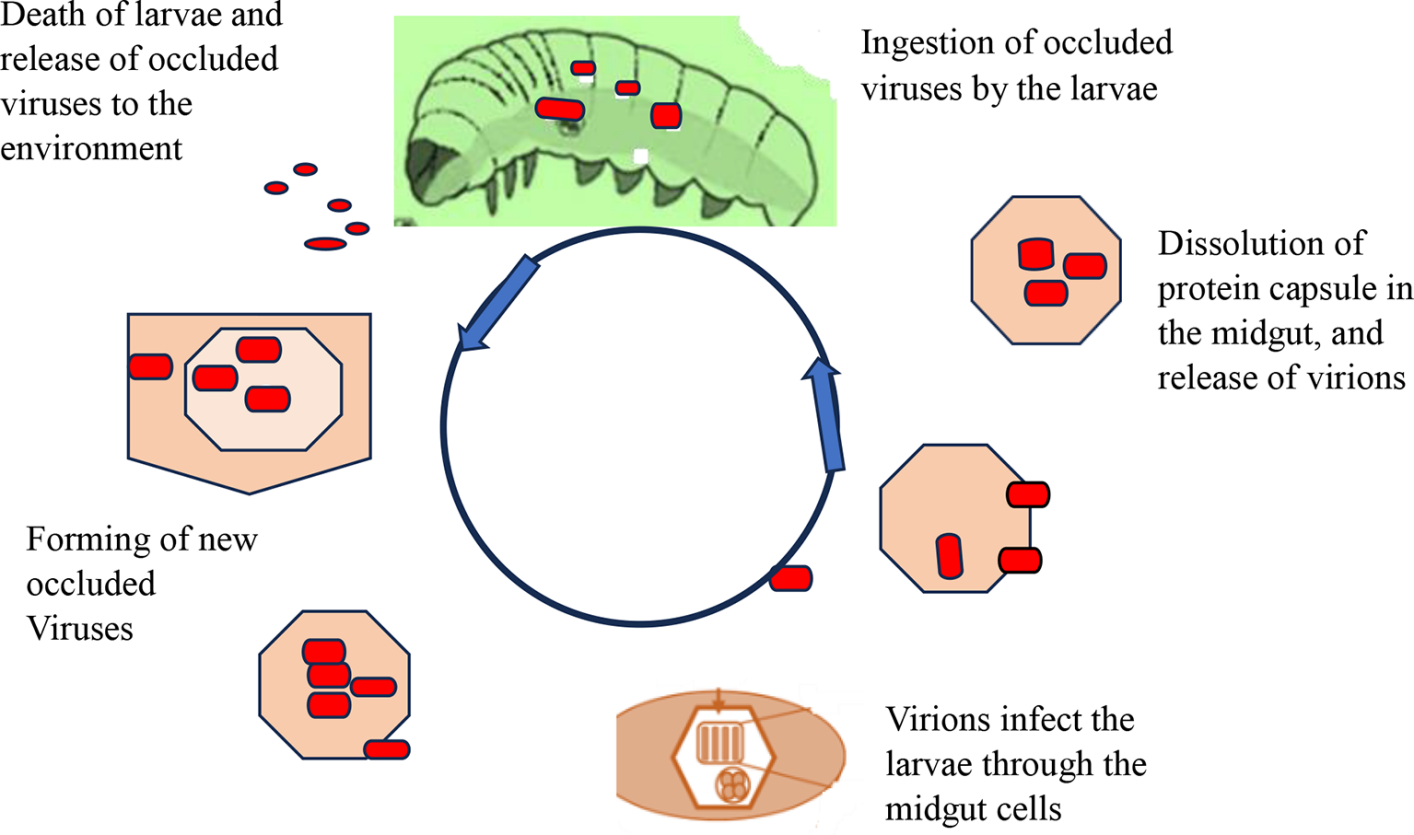
Mode of action of viral biopesticides. The virions enter the midgut cell nucleus, at which point the virus replicates within the nuclei of susceptible tissue cells, and tissue susceptibility varies greatly between viruses, with some NPVs being capable of infecting almost all tissue types and most GVs being tissue-specific replications. In the terminal stage of infection, the insect liquefies and thus releases polyhedral, which can infect other insects upon ingestion. A single caterpillar at its death may contain over 109 occlusion bodies from an initial dose of 1000. Under optimal conditions, target pests may be killed in 3-7 days, but when the condition is not suitable, death may be caused in 3-4 weeks ( Kalamkoff 2004).

Figure 4 shows how viral replication and infection take place and attack their target host. After infecting a target cell, viral replication in the nucleus or cytoplasm unfolds in three distinct phases: early (0-6 hours), second (6-24 hours), and very late (24-72 hours). During the late phase, the virus forms protective protein coats called occlusion bodies (Obs) or virions. These tiny packages containing multiple virus particles can lead to natural outbreaks (enzootics) that drastically reduce pest populations. However, sunlight poses a challenge: occlusion bodies can rapidly lose potency when exposed to ultraviolet radiation (UV) between 280-320 nm ( Killick 1990). Interestingly, studies show that plastic greenhouses can help mitigate this issue by filtering out over 90% of harmful UV-B rays, boosting infection rates in larvae ( Lasa et al. 2007).

The virions enter the midgut cell nucleus, at which point the virus replicates within the nuclei of susceptible tissue cells, and tissue susceptibility varies greatly between viruses, with some NPVs being capable of infecting almost all tissue types and most GVs being tissue-specific replications. In the terminal stage of infection, the insect liquefies and thus releases polyhedral, which can infect other insects upon ingestion. A single caterpillar at its death may contain over 109 occlusion bodies from an initial dose of 1000. Under optimal conditions, target pests may be killed in 3-7 days, but when the condition is not suitable, death may be caused in 3-4 weeks ( Kalamkoff 2004).

Several studies have been conducted to explore ways to improve the effectiveness of viral biopesticides. Certain formulations, like stilbene, can enhance susceptibility to NPV infection by disrupting the insect’s digestive barrier ( Okuno et al. 2003) or inducing cell death in the midgut ( Dougherty et al. 2006). Additionally, researchers are investigating genetically engineered viruses. For example, *vAcTaITX-1* and *vAcDTX9.2*, derived from specific spiders, show promise as commercial biopesticides against lepidopteran pests ( Hughes et al. 1997). While viral biopesticides offer numerous advantages over conventional chemical pesticides, including specificity and environmental safety, they face certain challenges for large-scale adoption. Producing them efficiently, particularly recombinant viruses, can be costly and labor-intensive, requiring specialized equipment and lengthy procedures ( Gupta 2010; Lacey et al. 2015). Despite these hurdles, organizations like IPM centers and state agricultural departments are actively supporting the development and small-scale production of these promising biopesticides.

*What is the main advantage of viral biopesticides*? Viral biopesticides offer a safer alternative, protecting human health, wildlife, and even beneficial insects. These targeted pathogens would not trigger resistance in pests, making them a lasting solution. Moreover, they seamlessly work with other pest control methods for a comprehensive defense ( Singh et al. 2019; Meenatchi and Aditi 2021). While viral biopesticides offer enticing, environmentally safe alternatives to chemical pesticides, they come with limitations. Unlike the broad spectrum of some conventional chemical insecticides, their use often remains targeted to specific pest species. Additionally, successful pest control can be a slow process, as infections may take a significant amount of time to become lethal and can be easily deactivated by high temperatures and ultraviolet radiation. Understanding these vulnerabilities is crucial for choosing the right pest control strategy ( Singh et al. 2019; Meenatchi and Aditi 2021) for the targeted pest and bioclimatic zone.

### 5.5 Nematodes as biopesticides

Entomopathogenic nematodes (EPNs) are classed as microbial pesticides, even though they are multicellular. EPNs are tiny soil-dwelling organisms that live in the water films around soil particles, fit nicely into IPM programs because they are considered nontoxic to humans, relatively specific to their target pests, and can be applied with standard pesticide equipment ( Shapiro-Ilan et al. 2006). These beneficial parasites help maintain the balance in the ecosystem by targeting specific pests without harming other organisms ( Kachhawa 2017). Two main families of EPNs, *Steinernematidae,* and *Heterorhabditidae*, have been enlisted in the fight against pests ( Bhat and Chaubey 2020). Species like *S. carpocapsae, S. thermophilum, H. bacteriophora,* and *H. indica* are commercially available and actively deployed in pest control programs in India ( Meenatchi and Aditi 2021). Commercial biopesticides based on EPNs contain non-feeding, third-stage infective juveniles (IJs) as the active ingredients. *Heterorhabditis* and *Steinernema* are mutualistically associated with bacteria of the genera *Photorhabdus* and *Xenorhabdus*, respectively ( Ferreira 2014).

**Figure 5.**
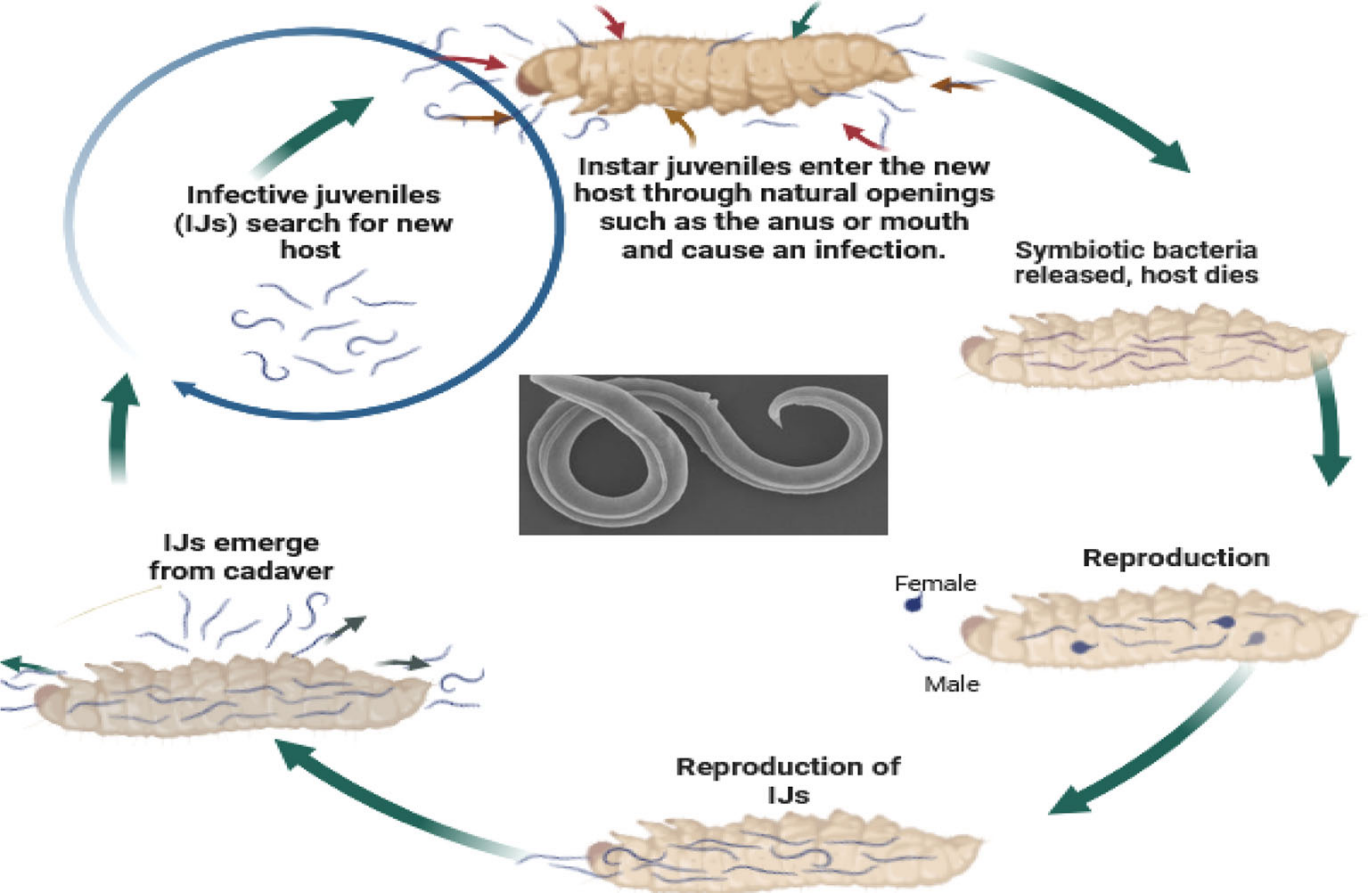
Mode of action of Entomopathogenic nematodes. The juvenile stage releases cells of their symbiotic bacteria from their intestines into the hemocoel. The bacteria multiply in the insect hemolymph, and the infected host usually dies within 24 to 48 hours. After the death of the host, nematodes continue to feed on the host tissue, mature, and reproduce ( Bedding 1982). The progeny nematodes develop through four juvenile stages to the adult. Depending on the available resources, one or more generations may occur within the host cadaver, and a large number of infective juveniles are eventually released into the environment to infect other hosts and continue their life cycle ( Bedding 1982). Nematodes enter the body cavity of insects and release their symbiotic bacteria into the host's intestine.

So, how do these tiny organisms take down their targets? EPNs, in their infective juvenile stage, barely millimeters long, use their keen senses to locate insects through carbon dioxide emissions, vibrations, and other chemical cues. They then wriggle their way into the insect’s body through natural openings like the mouth, anus, or breathing holes. Once inside, the nematodes release their symbiotic bacterial companions, setting off a natural pest control cascade. These bacteria produce toxins that quickly kill the insect within a week. The EPNs then feast on the insect’s liquefied remains, multiplying within the host to create new generations of tiny warriors. Finally, once their feast is over, the next generation of infective juveniles emerges, ready to seek out their next victim and continue the cycle ( Figure 5). Under ideal conditions, the impact of EPNs becomes visible within 5-7 days. Look for browning or tanning of insects infected by *Steinernematidae*, while those taken down by *Heterorhabditidae* turn a distinctive red ( Gupta 2010; Koul 2011; Ruiu 2018).

***Advantages of EPNs*:** EPNs offer a double win: safety and effectiveness. These organisms pose no threats to human health, plants, or animals, requiring no protective gear or waiting periods. Moreover, they leave no harmful residues, making them a sustainable choice for healthy crops and clean environments. Their target list reads like a bug buffet: cranberry girdlers, root weevils, webworms, and even wood borers tremble at their approach. However, these tiny heroes have their preferences. Moist soil around roots, protection from harsh UV, and short stints with other pest control methods are necessary for them to thrive, ensuring their targeted pest control doesn’t disrupt the natural ecosystem ( Ruiu 2018; Singh et al. 2019; Meenatchi and Aditi 2021).

***The disadvantages of EPNs*:** EPNs are highly susceptible to adverse weather conditions. Harsh UV rays and sizzling temperatures can quickly deactivate these tiny heroes, making their effectiveness dependent on fickle environmental conditions. Compared to their chemical counterparts, EPNs come with a hefty price tag. This, along with their short shelf life, makes them a less financially enticing option for some applications. EPNs can’t reach aerial pests, limiting their range of action. Additionally, relying solely on EPNs can disrupt the natural predator-prey balance, requiring repeated applications to maintain pest control. Furthermore, EPNs thrive in moisture. Pre- and post-irrigation, along with application during cooler hours, are essential for their success ( Meenatchi and Aditi 2021).

### 5.6 Protozoan biopesticides

Protozoans, also known as microsporidians, are intracellular parasites that can only survive by living inside other cells. They are found almost everywhere and can attack certain insects, like lepidopteran and orthopteran, making them useful in IPMs. Examples include *Nosema* sp. and *Vairimorpha* sp. However, despite their pest-specific nature and slow-acting properties, the use of protozoa as biopesticides is not as effective as other organisms, such as bacteria, viruses, and fungi. They can cause chronic and debilitating effects on their targets, but their success rate is lower compared to other biopesticides ( Meenatchi and Aditi 2021). Protozoans have a specific mode of action. Microsporidia infects the European corn borer, *Ostrinia nubilalis*, by being eaten by insects. The spores germinate in the midgut region, and the sporoplasm is then injected into the midgut cells. The spores then spread to different tissues and organs, multiply, and cause tissue breakdown and *septicaemia* ( Senthil-Nathan 2015).

## 6. Natural enemies of pests as biopesticides

Two powerful allies in this environmentally safe pest control approach are parasitoids and predators.

**Parasitoids**: These insects specialize in laying their eggs on or inside other insects. Their young hatch and feast on the host’s body from the inside out, eventually leading to the host’s demise. Examples include wasps that parasitize caterpillars and flies that target beetle larvae.

Parasitoids have a shorter life cycle than predators and can multiply at a faster rate. Therefore, they can be more effective in controlling insects. However, their presence may not be noticeable unless you examine samples of the insects to see if any adult parasitoids have emerged ( Yelitza et al. 2020).

**Figure 6.**
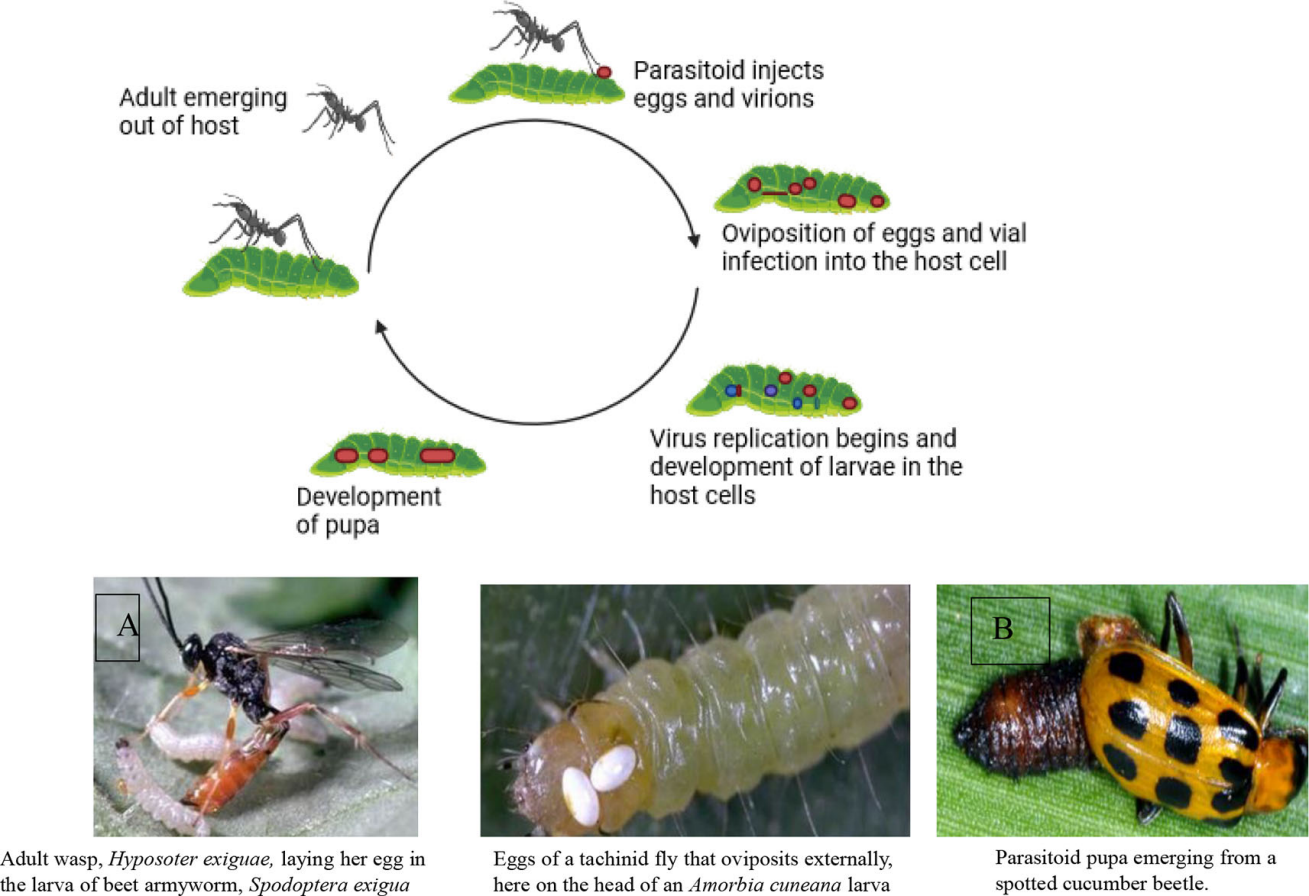
Natural enemies’ mode of action. A) Mode of action of parasitoids, and B) Natural predators (the insects are Lady beetles, (Coccinellidae), wasps, and fungus gnats, respectively).

**Predators**: These insects are fierce hunters who actively seek out and devour their prey. They’re often larger than their victims and use their speed, agility, and sharp senses to track them down. Ladybugs munch on aphids, dragonflies swoop on mosquitoes, and ground beetles patrol the soil for tasty treats. Introducing these natural predators into your environment creates a pest-fighting force on patrol. Figure 6 illustrates the mode of action of natural enemies in pest control, specifically focusing on A) the mode of action of parasitoids and B) natural predators, including lady beetles ( *Coccinellidae*), wasps, and fungus gnats.

Meenatchi and Aditi (2021) and Gautam (2008) have summarized several promising biopesticides that are effective against various crop pests. These specialized assassins are laser-guided for specific pests, efficiently hunting them even at low levels. They curb reproduction, keep infestations below damage thresholds, and play well with other pest-management tactics. Additionally, they help disrupt pest feeding and lessen the damage to your plants. They are environmentally safe pest control solutions ( Gautam 2008).

## 7. Biopesticide formulations

Biopesticide formulations, like conventional pesticide formulations, involve preparing a product from an active ingredient (biologically active metabolite or microbe) by adding various functional (active) and inert substances. This creates a product suitable for application to the target weed, pathogen, or insect using existing equipment. The active ingredient is combined with a carrier material and various additives to improve the biopesticide’s lifespan and efficacy ( Grewal and Peters 2005). Several factors significantly influence the economic viability of biopesticide products. These include the impact on the target pest, market size, pest spectrum, field performance consistency, production costs, and technological challenges (fermentation, formulation, and delivery systems), all of which pose potential hurdles to commercialization. Optimizing product formulations can significantly improve field performance consistency. This is crucial for the successful adoption and economic viability of many biopesticides ( Glass 1993). However, slow progress in research on formulation and delivery systems remains a major bottleneck for biopesticide development ( Lumsden et al. 1995).

The formulation is arguably the most crucial step in the development of biopesticides and is the cornerstone of product development ( Leggett et al. 2016). Ideally, a user-friendly formulation must meet several important criteria, including preserving and expressing the pesticidal properties of the microorganism, extending the shelf life to at least six months and preferably up to two years under ambient conditions, and enabling application using existing equipment ( Pusey 1994). Moreover, enhanced formulations can improve the efficacy of biopesticides by boosting their dispersion, attachment, and persistence at the target site ( Droby et al. 2009). Stickers improve adherence to foliage, enhancing persistence ( Schisler et al. 2004). Formulation stabilizes the biopesticide throughout production, distribution, storage, handling, and application to ensure persistence and activity at the target site. Cognizance must be given to ensure that the co-formulants used in biopesticide formulations are not chemical pesticide derivatives, are environmentally safe, and do not impact human health.

### 7.1 Biopesticide formulation requirements and challenges

The effectiveness of a biopesticide hinges on the biological activity of its active microorganism or metabolite. In its raw state, this active ingredient requires formulation to enable its practical handling, storage, and application ( Harald 2019). A crucial distinction between conventional (chemical) pesticide formulation and biopesticide formulation lies in the biopesticide’s living nature. Biopesticides are sensitive to storage conditions and environmental factors, necessitating careful formulation to preserve their biological viability ( Harald 2019). Formulating biopesticides faces several challenges, including ensuring market viability, ease of production and application, product stability during storage and transport, and long-term viability and efficacy of the biopesticide ( Lumsden et al. 1995). Contributing factors to their limited commercial success include production difficulties, sensitivity to environmental stressors, and lack of appropriate formulation ( Powell and Jutsum 1993). Another challenge is the slime layer produced by gram-positive bacteria like *Bacillus spp.* during endospore formation. This slime can clog sprayer filters and nozzles, hindering application. While removing the slime through methods like centrifugation is possible, it may also remove valuable components contributing to the biopesticide’s effect ( Boyette et al. 1996).

The goal of formulation is to improve product stability, bioactivity, delivery, and integration into pest management systems. Successful formulations also prioritize user convenience, compatibility with equipment and practices, and effectiveness at practical rates. Environmental factors like temperature, moisture, and UV exposure significantly influence efficacy, particularly for foliar-applied biopesticides ( Alan 1988). Soil properties, moisture, temperature regimes, and microbial competition impact soil-applied biopesticides. All these factors need careful consideration during formulation development.

### 7.2 Types of biopesticide formulations

**7.2.1 Formulation of bacteria biopesticide**

Several recent studies highlight the growing use of bacteria-based biofungicide formulations as a safe and effective complement to conventional chemical pesticides ( Cook 1993; Lumsden et al. 1995; Elad 1995). *Bacillus*-based products are already boosting crop yields in various countries, from China to the United States ( Zhang et al. 1996). These wettable powders, compatible with existing fungicides, target diseases caused by *Rhizoctonia* and *Fusarium* in a range of crops like cotton, legumes, vegetables, and ornamentals.

Mass production of these biofungicide bacteria formulations typically involves deep-tank liquid fermentation. However, in some cases, methods like semisolid or solid-state fermentation might be more suitable. Key factors influencing both bacterial growth and their beneficial metabolite production include the medium’s nutrient composition and optimal growth conditions ( Paau 1988). Cost-effectiveness and readily available components are prioritized. The final formulation, usually involving additional ingredients after fermentation, comes in various forms like solids, slurries, powders, or granules. Long-term viability during transport and storage (at least 4 months) is crucial ( Paau 1988). Formulation significantly impacts the biofungicide’s performance by enhancing bacterial survival after application. This involves creating a protective environment that maximizes the bacteria’s potential for successful colonization and disease control. Choosing the right formulation can help ensure consistent results in the field.

The products are designed as wettable powders and can be used with various seed treatment fungicides ( Table 1). *Agrobactenum tumefaciens* is available commercially in Australia, the United States, and New Zealand and is formulated as a concentrated liquid or moist peat-based product or supplied as a non-formulated agar culture ( Kerr 1980; Elad 1995). After being suspended in water, the bacteria can be applied to the seeds, cuttings, roots, or root wounds of susceptible orchards and ornamental plants as a dip, spray, or drench. *Agrobacterium tumefaciens*, available as a liquid, peat-based product, or agar culture, combats diseases in orchards and ornamental plants through dips, sprays, and drenches. A strain of *A. radiobacter* known as K84 is used as a biopesticide to control crown gall disease ( Stockwell & Stack 2007). *Streptomyces griseoviridis* (Mycostop), formulated as a wettable powder, tackles damping-off and root rot in vegetables and ornamentals caused by *Fusarium*, *Phomopsis,* and *Pythium.* It can be applied dry to seeds or as a liquid drench and is compatible with chemical pesticides ( Muir 2023). Three *Burkholderia assian* strains (Blue Circle, Deny, and Intercept) come in liquid and fight a wider range of enemies. These bacterial warriors target fungi like *Fusarium, Phytophthora, and Pythium* alongside nematodes, including *Globodera rostochensis, Heterodera glycines,* and *Hoplolaimus columbus* ( Kumari et al. 2022) *.*

Most commercial bioinsecticide formulations today harness the power of *Bacillus thuringiensis* ( *Bt*), a friendly, spore-forming bacterium. This gram-positive warrior wields a powerful weapon: delta-endotoxin proteins that wreak havoc on the mid-guts of susceptible insect larvae ( Cannon 1993) ( Table 1). *Bt* proteins are highly specific, only affecting a few species in the Lepidoptera (butterflies and moths), Coleoptera (beetles), and Diptera (flies) families. *Bt* biopesticides come in an array of formulations, from concentrated liquids to handy dusts, catering to different application needs.

**7.2.2 Formulation of fungal biopesticides**

Fungicide formulations primarily include *filamentous fungi* (e.g. *Gliocladium virens* and *Trichoderma harzzanum*) *,* yeast-like fungi (e.g *. Pseudozymajlocculosa* ( Bélanger 1997) and *Tllletiopsis pallescens* ( Urquhart and Punja 1997) *.* These biofungicides are used to control root-infecting pathogens (e.g. *Pythlum, Rhizoctonia)*, and foliar fungal pathogens (e.g. powdery mildew ( Bélanger 1997; Urquhart and Punja 1997; Punja 1997) and *Botrytzs* ( Elad 1995). Environmental conditions, such as temperature and moisture, can impact the growth and survival of fungal biofungicides. Various formulations have been developed for the application of spore inocula, including granules, pellets, dust, and wettable powders. These formulations can be applied directly or suspended in water or oil, as is the case with *Cylindobasidium leave* ( Muir 2023) *.*

Granular formulations not only protect against desiccation but also offer a food base for the fungus. Meanwhile, powders can be easily sprayed and can provide coverage for large areas. An example of this is the large-scale aerial application of sawdust covered in *Colletotrichum acutatum* spores for the control of *Hakea sericea* ( Jacobson and Azevedo 2023). It is also possible to treat seeds with liquids or dust for the application of these biopesticides. Moreover, spore formulations in inverted emulsions have been tested for yeasts, such as *Tilletiopsi*s ( Urquhart and Punja 1997).

The use of alginate prill has been successfully developed to create a granular formulation of *Gliocladium virens* (Soil Gard), which helps control root-infecting fungi in potting media ( Lumsden et al. 1995). Similarly, *Trichoderma* powder or dust formulations with pyrophyllite clay (Pyrax) have also been proven to be effective ( Jayaraj and Radhakrishnan 2006). To produce the necessary biomass, appropriate nutrient substrates are used in large-scale deep tank fermenters. The resulting product can either be used wet or dried before formulation ( Lumsden et al. 1995). Most of the factors that affect product development for fungi are like those for bacteria.

Entomopathogenic fungi are a fascinating group of soil-dwelling microorganisms that play a crucial role in biological control. These fungi are capable of infecting and killing insects through cuticle penetration. They don’t need to be ingested by the insect hosts; instead, they directly invade through the insect’s outer covering. This unique mode of action allows them to control a wide range of insect pests, including sucking insects. However, they cannot effectively combat viruses and bacteria similarly ( Shishupala 2022). Entomopathogenic fungi are a type of fungi that can target and control pests without harming beneficial organisms such as pollinators. They are an environmentally safe alternative to traditional chemical pesticides for managing agricultural pests. Some of the most well-researched fungal species with bioinsecticidal properties include *Akanthomyces muscarius, Beauveria bassiana, Cordyceps fumosorosea, Purpureocillium lilacinum, Verticillium*, and the *Metarhizium anisopliae* species complex ( Shishupala 2022). A recent study by Luo *et al*. (2022) isolated a new entomopathogenic fungus from an adult cadaver of *D. citri.* The fungus was identified as *Cordyceps fumosorosea* based on its morphology and ITS sequence analysis. The researchers named this specific isolated *C. fumosorosea SCAU-CFDC01.* The study assessed the pathogenicity of the strain against *D. citri* nymphs and adults in both laboratory and greenhouse settings.

Various fungi can be used to control different types of insects. *Verticillium lecanil* can control aphids, *Beauveria bassiana* can control whiteflies, locusts, and beetles, *Metarhizium anisopliae* and *M. flavoviride* can control mosquitos and locusts respectively, while *Lagenidium gigantem* can control mosquito larvae ( Lacey and Goettel 1995; Bateman 1997; FAO 2017; Savita 2019). These fungi can be applied to insects in different ways, such as wettable powders, emulsions, specks of dust, baits, or traps, or even added to the soil ( Auld 1992; Feng et al. 1994; Goettel et al. 1995; Inglis et al. 1996) or used in indoor residual spraying. It is important to formulate these fungi to protect against environmental conditions such as moisture, temperature, UV damage, and desiccation. For example, *B. bassiana* conidia on foliage can be damaged by UV-B radiation, which is part of the sunlight spectrum ( Caudwell and Gatehouse 1996a; Daoust and Pereirn 1986). In field conditions, these entomopathogens can be applied at ultralow volumes (ULV) to increase their effectiveness and protect against UV damage ( McGUIRE and Shasha 1992; Moore et al. 1993; Inglis et al. 1996). To improve survival and shelf-life, sunlight blockers (such as clay) or UV-B absorbing compounds (such as Tinopal) can be added to inoculum formulations or starch encapsulation ( Pereira and Roberts 1991; McGuire and Shasha 1992; Caudwell and Gatehouse 1996b).

**7.2.3 Formulation of mycoherbicides**

Environmental factors, such as temperature and moisture, can greatly impact the effectiveness of Mycoherbicides. The moisture needed for the disease to develop is often determined by the amount of dew that occurs. “DeVine” is the first registered Mycoherbicide, which is a liquid formulation consisting of chlamydospores of *Phytophthora palmivora* ( Burnett et al. 1974; TeBeest and Templeton 1985). This product has a limited shelf life of only 6 weeks when refrigerated due to its instability. Another Mycoherbicide called “Collego” ( *Colletotrichum gloeosporioides f.sp. aeschynomene*) is formulated as dried spores and is available in a wettable powder ( Boyette 1994). The commercialized formulation of *Colletotrichum acutatum* is formulated as dried spores that are mixed with water when ready to apply, and Stumpout is *Cylindrobasidium laeve* formulated as dried spores in an iron carrier that is mixed with standard cooking oil when ready to apply ( Muir 2023).

There are several ways to improve the effectiveness of mycoherbicides, which are used to control weeds ( Boyette et al. 1996). One way is to use adjuvants and other additives that can help spores grow, make the pathogen more stable, change the environment, or make it possible to use bioherbicides on more plants ( Boyette 1994). For example, a fungus called *Colletotrichum truncatum* can kill a weed called hemp sesbania, but it needs a lot of moisture to work ( Boyette et al. 1993). Adding unrefined corn oil to the biocontrol agent can help it work better, so less moisture is needed. This makes it easier to use the bioherbicide and reduces the amount of spray needed ( Boyette 1994).

Surfactants are ingredients used in formulations to help wet plants by reducing surface tension and improving the dispersal of fungal spores in spray droplets. Some surfactants used include Tween 20 with *Fusarlum lateritum*, nonoxynol with *Alternaria macrospora* and *A. assia*, and *sorbitol* with *Colletotrichum coccodes* ( Boyette 1994). However, evaluating the inhibitory or stimulatory effects on spore-germination and infection is important before selecting appropriate surfactants and ensuring that the surfactants do not contain pesticide derivatives. Water-in-oil invert emulsions have been used with foliar fungal biopesticides to provide a favorable environment for germination and infection ( Connick et al. 1990; Daigle et al. 1990; Auld and Morin 1993; Boyette et al. 1996). Using inverted emulsions ( Boyette et al. 1993) has been found significantly to improve the efficacy of *C. truncatum* and *Alternaria assia.* However, inverted emulsions are very viscous and may demonstrate phytotoxicity in some target plants ( Walker and Boyette 1985; Amsellem et al. 1990; Goettel et al. 1995). Connick et al. (1990) developed an inverted emulsion with improved water-retention properties that was less viscous. Additionally, vegetable oils can enhance the efficacy of mycoherbicides, such as *Colletotrichum orbiculare*, for the control of spiny cocklebur ( Auld 1992) and *Collototrichum acutatum* for the control of *Hakea sericea* and *H. gibbose.* No phytotoxicity and improvements in the spread of the inverted emulsion were observed.

Solid-based formulations of mycoherbicides have been developed for weeds that are infected at or below the soil surface, which is more suitable for preemergence mycoherbicides ( Daigle and Connick 1990; Boyette et al. 1996). These formulations can provide a food base for the pathogen, act as a buffer in extreme environmental conditions, and retain inoculum that may not be easily washed away. One such formulation, known as “PESTA,” uses a wheat-gluten matrix consisting of liquid inoculum, semolina wheat flour, and kaolin. This formulation includes fungal agents like *C. truncatum, A. crassa,* and *Fusarium lateritium* and can be applied as preemergent and soil-incorporated treatments ( Boyette et al. 1984). The shelf life of the product can be improved by manipulating the water activity and sucrose content. Other solid substrates used to formulate mycoherbicides include bran, wheat kernels, cornmeal/sand, vermiculite ( Auld and Morin 1993; Boyette et al. 1996), and sawdust ( Muir 2023). For example, cornmeal/cornmeal sand was used to formulate mycelium, micro- and macroconidia, and chlamydospores of *Fusarium solani f.sp. cucurbitae* for the control of Texas gourd. This pre-emergent granular formulation provided 96% control of the weed ( Boyette et al. 1984).

**7.2.4 Formulation of viruses**

Baculoviruses have been studied as a potential solution for controlling insect pests that belong to the Lepidoptera, Hymenoptera, and Coleoptera families ( Gory and Bishop 1995). These viruses have several advantages, such as high specificity, not harming beneficial insects, and persisting in the environment, which makes long-term control of insect pests possible without damaging the environment. Nuclear polyhedrosis viruses (NPVs) and granulosis viruses (GVs) are examples of baculoviruses. However, there are some limitations to using these biopesticides, such as their slow speed of biological activity, low stability under UV light, and difficulties in production ( Powell and Jutsum 1993).

The stability of the baculoviruses is often a function of viability, but it is not a significant problem for small-scale field trials as the viruses can be collected from macerated larvae, mixed with water, and stored for short periods through refrigeration. However, these systems are not suitable for large-scale production and application ( Gory and Bishop 1995). The formulation of these viruses is an important aspect of product development, but researchers have not given it as much attention as bacteria and fungi.

Baculoviruses are usually available in the form of concentrated wettable powders. The biocontrol product called “Elcar” for corn earworm ( *Helicoverpa zea*) NPV can be spray-dried or air-dried after being diluted with an inert carrier. The gypsy moth ( *Lymantria dispar* L.) NPV is freeze-dried either with carbohydrates or by acetone precipitation ( Gory and Bishop 1995). The virus can be inactivated by UV radiation, especially wavelengths of 290-320 nm. UV protectants like reflections or absorbers can be added to formulations to stabilize baculoviruses. Several dyes, such as lissamine green, acridine yellow, alkali blue, and mercurochrome, have been used as UV protectants, especially to absorb UV-A irradiation ( Shapiro 1995). Optical brighteners (fluorescent brighteners), which are commonly used in soaps, detergents, and fabric softeners, also absorb UV light. They have been shown to significantly reduce the photodegradation of NPVs and enhance viral activity ( Shapiro 1992; Dougherty et al. 1995; Shapiro 1995). Care must be taken to ensure that these optical brighteners do not adversely affect the environment due to residues and residuality.

**Figure 7.**
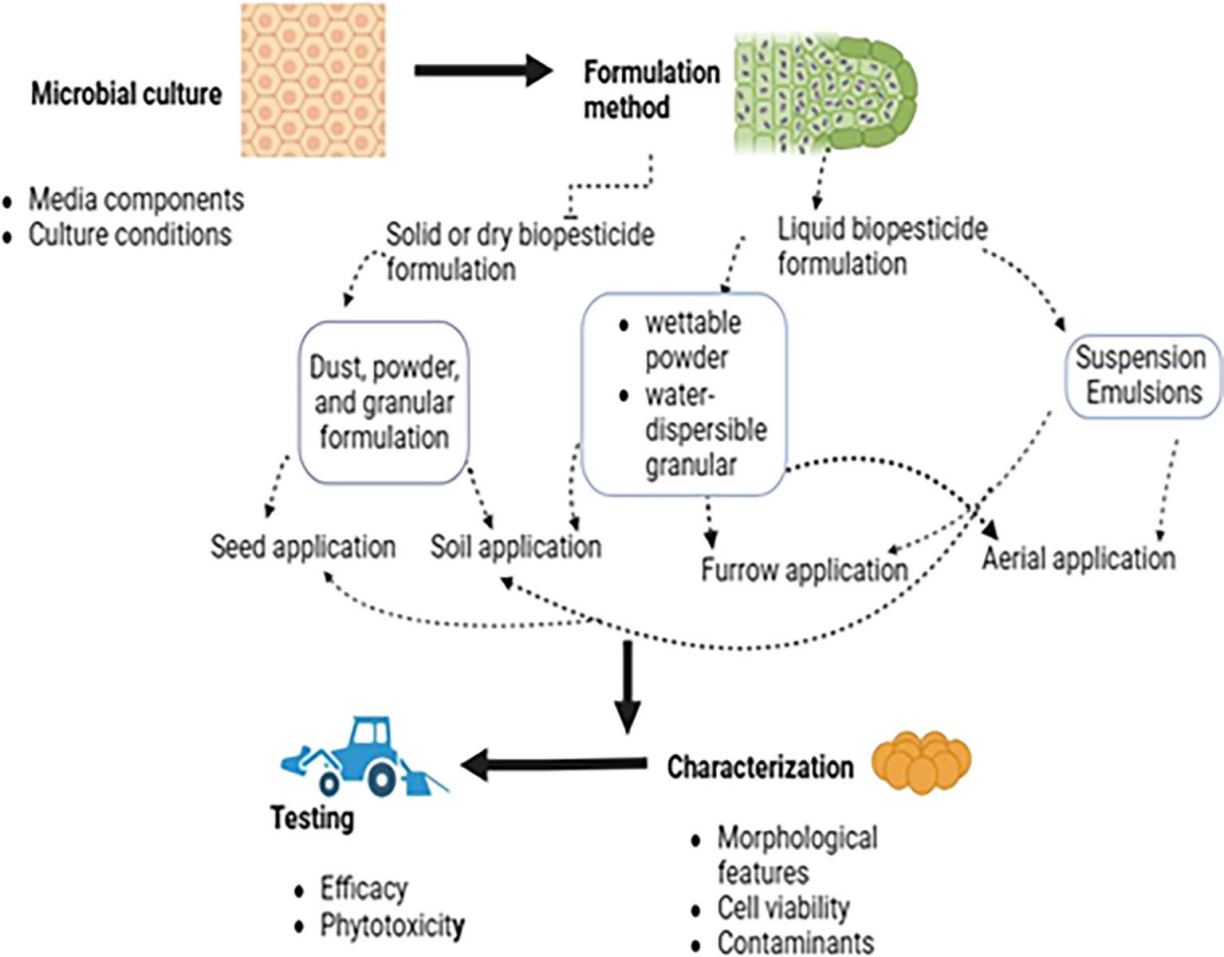
General workflow of the procedures involved in the development of microbial biocontrol formulation. Straight line arrows indicate process (steps), and broken lines indicate categories or classifications.

There are two types of biopesticide formulations: liquid and dry, which differ based on their physical state ( Figure 7). Dry formulations (dust powders, granules, seed dressers, wettable powders, and wettable) dispersible powder. They are produced using various technologies, including spray drying, freeze drying, or air drying, with or without a fluidized bed ( Li 2002). They typically require binders, dispersants, and wetting agents for optimal efficacy and application. Liquid formulations can be water-based, oil-based, polymer-based, or combinations. Water-based formulations, like suspension concentrates, suspo-emulsions, capsule suspensions, and ultra-low volume liquids, require additional inert ingredients for stability and performance ( Boyetchko et al. 1999). These include stabilizers, stickers, surfactants, coloring agents, antifreeze compounds, and additional nutrients. Care must be taken to ensure that these inert ingredients do not adversely affect the environment due to residues and residuality and do not contain pesticide derivatives.

## 8. Advances in biopesticide and delivery technologies enhance the efficacy of biological products

Advances in biopesticide application techniques have been driven by the need for more efficient and targeted pest control methods while minimizing environmental impacts and fate. These advancements aim to improve the delivery, efficacy, and performance of biopesticides. Several improved spray technologies and precision targeting methods have been developed, including controlled droplet application, variable rate technology, intelligent spray systems, air-assisted sprayers, robotics, and automation technologies, automated targeting systems ( Wang et al. 2019), thermal fogging, and aerial application. The improved spray technologies play an important role in delivering droplets of consistent size and velocity, improving spray deposition, reducing non-target drift, optimizing chemical pesticide usage, reducing costs, and minimizing environmental impacts and fate. Additionally, some of these technologies utilize sensors and algorithms to detect and target pests in real-time, enabling precise application of biopesticides only where needed. Other techniques improve the effectiveness of biopesticides by reaching target areas that are otherwise difficult to treat, and some spray techniques improve coverage and reduce the amount of biopesticide needed.

### 8.1 Seed treatments and soil applications

Seed treatments and soil applications involve various methods for applying biopesticides to protect seeds and enhance crop establishment. Seed coatings and treatments apply biopesticides onto the seed surface, providing early protection and improving crop establishment ( Khan et al. 2020). Incorporating biopesticides into soil amendments can improve soil health, control soil-borne pests, and enhance plant growth, offering sustainable pest management and environmental benefits. Other methods include soil drenching, which involves applying biopesticides as a liquid solution directly to the soil around the plant base. This technique targets pests in the soil, such as nematodes and soil-borne pathogens. Biopesticide-based soil fumigation utilizes the controlled release of compounds to control pests and diseases in the soil, providing an alternative to chemical fumigation but with reduced environmental impacts. Rhizosphere application focuses on applying biopesticides directly to the root zone of plants. This technique targets pests that interact with plant roots, such as soil-dwelling insects and nematodes. These seed treatment and soil application methods offer effective pest management while minimizing environmental impacts and fate.

### 8.2 Factors affecting the efficacy of biopesticides

Various challenges and limitations, including environmental conditions such as temperature, humidity, and sunlight, can influence by the efficacy of biopesticides during pest control. Some biopesticides may require specific temperatures or moisture levels for optimal performance. Additionally, biopesticides often exhibit narrow target specificity that is effective against specific pests but not others. This limitation reduces their broad-spectrum efficacy, and it may be necessary to use multiple biopesticides to target different pests ( Gurr et al. 2017). Furthermore, the formulation and storage conditions of biopesticides can affect their stability and shelf life. Factors such as pH, temperature, and exposure to light can degrade the effectiveness of biopesticides over time ( Riga et al. 2017).

It’s important to note that these challenges and limitations can vary depending on the specific biopesticide and pest control scenario. By considering and addressing these factors, it is possible to optimize the efficacy of biopesticides in pest control practices. The efficacy of biopesticides can be influenced by the application methods used. Factors such as spray volume, droplet size, and coverage play a crucial role in the distribution and effectiveness of the biopesticide on the target pests ( Chandler et al. 2011). Ensuring that the application method is properly calibrated is important to achieve adequate coverage and contact with the pests ( International Symposium on Food Safety and Control).

Biopesticides may have a shorter persistence compared to chemical pesticides, which may require more frequent applications. Their residual activity on plants or the environment can be limited, necessitating careful timing and repeated treatments. Proper timing of application is essential to target pests during vulnerable stages of their life cycle thus, it is critical to fully understand the pest’s lifecycle before implementing a biopesticide programme. In addition to the challenges mentioned earlier, several other factors can limit the efficiency of biopesticides. These include regulatory approval, which can be a complex and time-consuming process. Limited availability and accessibility of biopesticides can pose challenges for farmers ( Biondi et al. 2021). And their compatibility with integrated pest management can be a complex issue. Lack of standardization and quality control protocols may affect the consistent performance of biopesticides. Furthermore, biopesticides can be more expensive compared to chemical pesticides, affecting their cost-effectiveness. The efficacy of biopesticides can vary depending on factors such as pest population density, pest development stage, and resistance mechanisms. Pests can develop resistance to specific biopesticides over time, which can reduce their effectiveness. Therefore, it is important to monitor pest populations and implement IPM strategies that incorporate the use of different biopesticides and other pest control measures to mitigate the development of resistance.

## 9. Future perspectives and research directions

**Figure 8.**
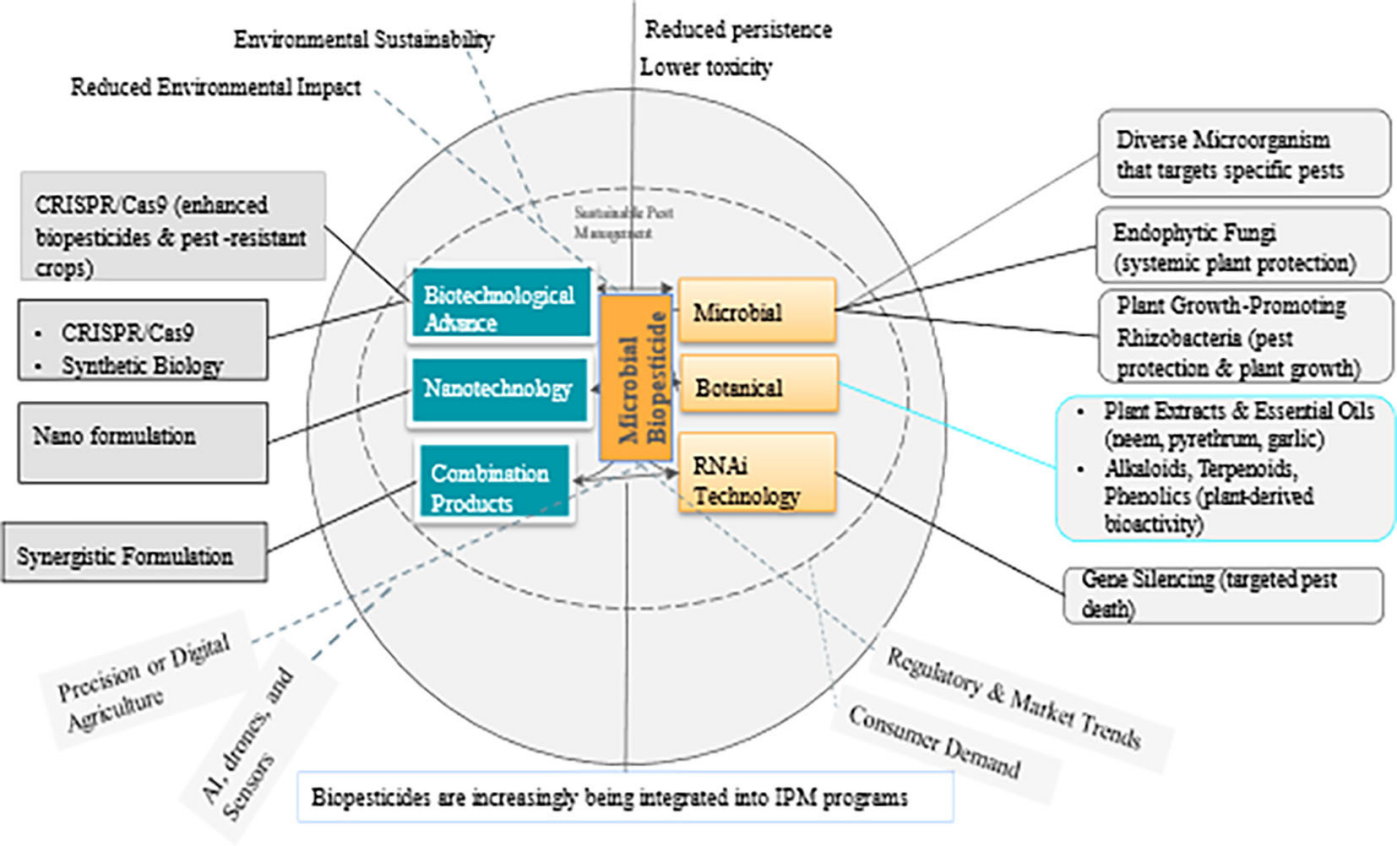
Summary of the emerging trends in biopesticide development.

Emerging trends in biopesticide development are driven by the need for sustainable agricultural practices and the increasing demand for ecologically safe pest control methods. Some of the key trends are summarized in Figure 8. Future perspectives and research directions in the field of microbial biopesticides including key research directions include Genetic engineering to improve microbial agents, synergistic interactions with other pest control methods, advanced formulation and delivery techniques, resistance management strategies, environmental impact assessments, integration into sustainable agriculture, and public awareness and adoption are essential for advancing their development and application. The future of microbial biopesticides looks promising, with ongoing research focusing on enhancing their efficacy, specificity, and environmental safety ( Karlsson et al. 2020). Microbiopesticides are gaining significant attention in modern agriculture due to their environmentally safe nature and reduced impact on human health. Researchers and companies are actively involved in developing new biopesticide formulations and strategies to enhance their efficacy. Some emerging trends in biopesticide development include microbial biopesticides, plant extracts and essential oils, RNA-based biopesticides, nanotechnology in biopesticides, and the combination of different biopesticides for improved pest control ( Karlsson et al. 2020). As the demand for sustainable and ecologically safe pest management strategies has increased, biopesticides have made tremendous strides in recent years. One of the most significant discoveries is the use of microbial biopesticides, such as bacteria, fungi, and viruses, with qualities enhanced through genetic modification or natural selection. By combining CRISPR/Cas9 and RNAi, researchers can develop microbial biopesticides with improved specificity, efficacy, and environmental safety. These technologies offer exciting possibilities for sustainable agriculture. Figure 8 provides a comprehensive summary of the emerging trends in biopesticide development and the integration of biopesticides with other integrated pest management (IPM) practices and pest control methods.

These trends indicate a growing focus on developing innovative, effective, and sustainable pest control solutions that align with environmental conservation and agricultural productivity goals.

## 10. Conclusion

The adoption of biopesticides is an increasingly popular trend in agriculture due to various environmental, health, and economic benefits. Biopesticides consist of naturally occurring substances, including insects, nematodes, microorganisms, plants, and semiochemicals, along with their by-products, offering a sustainable alternative to traditional chemical pesticides. Despite the benefits, several challenges can hinder the widespread adoption of biopesticides. Novel microbial strain creation with improved features, such as greater efficacy and larger target spectra, should be prioritized in future research and the use of biopesticides. To maximize their efficiency and reduce the possibility of resistance, biopesticides’ modes of action must be understood. Along with other tactics, using biopesticides in complete pest management programs can improve overall pest control. The stability, adhesion, and targeted distribution of biopesticides will be improved through improvements in formulation and delivery techniques. Risk assessment and regulatory frameworks must be created to guarantee the safety and effectiveness of biopesticides. To encourage knowledge and acceptance of biopesticides, efforts should be made to educate and raise public awareness. Collaboration and knowledge sharing among researchers, stakeholders, and regulatory agencies will facilitate progress in the field. By focusing on these recommendations, biopesticides can contribute to sustainable and environmentally safe pest management practices in agriculture.

## Authors contribution

Conceptualization: K.T.M.; Original draft preparation: K.T.M. and L.M.; Writing: K.T.M., D. M.; and D.M.; Editing, Reviewing, and formatting: D.N.: and Reviewed drafts of the paper: J.G.; Language editing and preparation of tables and/or figures: K.T.M. and D.N.; Final approval of the review to be published.

All authors have read and agreed to the published version of the manuscript.

## Data Availability

No data are associated with this article.
