# Supplementary material for: Recent Advances in Biopesticide Research and Development with a Focus on Microbials
Source: F1000Res. 2025 Mar 11;13:1071. Originally published 2024 Sep 19. [Version 5] doi: 10.12688/f1000research.154392.5 (PMC11541078; doi:10.12688/f1000research.154392.5)
Supplement: Peer review history

# Reviewer report: Reviewer response for version 4

**DOI:** 10.5256/f1000research.177450.r365526

**Published:** 2025-02-19

**Authors:** Alessandro Mattedi

**Affiliations:** 1 University of L’Aquila, Coppito, L’Aquila, Italy

Thank you for your suggestions. We have included the following references.

- Abdallah, Dorra Ben, Slim Tounsi, Houda Gharsallah, Adnane Hammami, and Olfa Frikha-Gargouri. "Lipopeptides from Bacillus amyloliquefaciens strain 32a as promising biocontrol compounds against the plant pathogen Agrobacterium tumefaciens." *Environmental Science and Pollution Research* 25 (2018): 36518-36529.
- Brown, Pamela JB, Jeff H. Chang, and Clay Fuqua. "Agrobacterium tumefaciens: a transformative agent for fundamental insights into host-microbe interactions, genome biology, chemical signaling, and cell biology." *Journal of Bacteriology* 205, no. 4 (2023): e00005-23.
- Mnif, Inès, and Dhouha Ghribi. "Potential of bacterial derived biopesticides in pest management." *Crop Protection* 77 (2015): 52-64."

**Reply: The cited papers do not talk about the biopesticide properties of *Agrobacterium tumefaciens* that the manuscript mentions, and you did not explain in what they consist. Please verify whether they are the correct references.**

 "14) " *Agrobactenum tumefaciens* is available commercially in Australia, the United States, and New Zealand"

 What about other countries?

 Please also give examples of commercial biopesticide products containing A. tumefaciens.

- Response: Thank you for your suggestions There are no recent *A. tumefaciens* products"

**Reply: If there are no products available, then how it is possible that it is "available commercially" in the mentioned countries as a biopesticide? Please clarify this section.**

 "15) I also recommend the possibility to mention Agrobacterium radiobacter and how it is sometimes confused.

- Response: Thank you for your suggestions and comments. A strain of *A. radiobacter* known as K84 is used as a biopesticide to control crown gall disease (Stockwell et al. 1993).

Stockwell, V. O., L. W. Moore, and J. E. Loper. "Fate of Agrobacterium radiobacter K84 in the environment." *Applied and Environmental Microbiology* 59, no. 7 (1993): 2112-2120."

**Reply: Perhaps due to an oversight, in the manuscript you added the wrong reference (Stockwell & Stack, 2007), plus you did not mention how the species is sometimes confused. Please check.**

Is the review written in accessible language?

Partly

Are all factual statements correct and adequately supported by citations?

Partly

Are the conclusions drawn appropriate in the context of the current research literature?

Partly

Is the topic of the review discussed comprehensively in the context of the current literature?

Partly

Reviewer Expertise:

Environmental microbiology

I confirm that I have read this submission and believe that I have an appropriate level of expertise to confirm that it is of an acceptable scientific standard, however I have significant reservations, as outlined above.

# Reviewer report: Reviewer response for version 3

**DOI:** 10.5256/f1000research.174749.r351244

**Published:** 2025-01-11

**Authors:** Alessandro Mattedi

**Affiliations:** 1 University of L’Aquila, Coppito, L’Aquila, Italy

Thanks to the authors for writing a long review about this topic. I have some minor corrections and suggestions about the manuscript. Please forgive me in advance if my language is not clear enough or heavy to read:

 1) The review in general could benefit from a more synthetic language. Often, paragraphs can be shortened while conveying the same amount of information. Occasionally, it also happens that the same concepts are repeated, for example when illustrating the several advantages of biopesticides or how they align with several different target strategies. There are other examples that could benefit from reading again the text and summarizing repetitions. I suggest the authors to consider this possibility, as it can help to promote their ideas in a more effective and concise way. 

 2) "However, resistance to some *Bt* products has been noted."

 I recommend to add a brief mention on how and why this might have happened.

 3) It could be worthy to note that recently some concerns have arisen about the possibility that Bt toxins could be involved in a fraction of foodborne outbreaks, although there are still relevant gaps in knowledge that needs to be filled and we cannot yet draw definitive conclusions. Still, the reader might be interested in pursuing further studying and investigation of the topic. A reference, Ref (1)

 Not strictly necessary, depending on the goals.

 4) There are other entomopathogenic bacteria of interest such as Lysinibacillus sphaericus, Brevibacillus laterosporus, Serratia marcescens, Yersinia entomophaga etc.. The topic is much larger and has quite interesting considerations regarding the feasibility of applications, their effectiveness, and the large-scale limitations. For example: Ref (2)

 5) "Besides, biopesticides 1) generate fewer greenhouse gasses (GHG) than chemical pesticides, 2) improve nutrient availability for plants, 3) increase soil fertility, and 4) offer a more environmentally safe approach to pest control in agriculture, which can be useful in the fight against climate change."

 Please provide references for these points.

 Please elaborate points 2 and 3: if you are stating that biopesticides per se are also biostimulants, this requires a better explanation.

 Please expand point 4 or condensate it with point 1.

 6) "Microbial biopesticides offer diverse delivery methods to effectively reach crop targets. These methods include live organisms, dead organisms, and spores"

 Please describe these methods here for a better understanding of the scientific mechanisms.

 7) "Additionally, they leave no harmful residues, don’t harm pollinators and other valuable organisms, and can be applied close to harvest time."

 This is actually a slight approximation. Recent research show that there could be some negative effects after all, and that there are knowledge gaps regarding pollinators. It is always a matter of balancing risks and benefits. While the low impact status of biopesticides is not changed, it is still worth to remember peculiarities and potential limits. For reference: Ref (3)

 8) "Bacterial biopesticides, while effective, come with a few disadvantages. For instance, they only target specific species or groups of insects, leaving other pests to survive and continue causing damage. "

 This is conceptually a bit clashing with the previous statement that specificity is an advantage, in order to avoid non-target insects. It is better to reformulate in a clearer way that the specificity gives that advantages but requires complementarity of treatment when dealing with different pests.

 9) The review doesn't adhere to the same structure within its sections. For example, section 5.5 gives two explicit separated paragraphs titled "advantages" and "disadvantages" of EPNs, while other sections discuss them as part of the flow in the text. I suggest to uniform the style for better clarity and readibility.

 10) Section 6 is a bit superficial and doesn't mention the need to assess that alloctonous predator can be safely controlled - i.e. they cannot become invasive. Since the review focuses on microbials, it doesn't need to go in detail about macrobials, but better accuracy is required.

 11) Some phrases like "reduce chomping on your precious plants", "This gram-positive warrior wields a powerful weapon: delta-endotoxin proteins that wreak havoc on the mid-guts of susceptible insect larvae" or "These technologies offer exciting possibilities for sustainable agriculture" sound quite odd and out of place for a technical review. Are they referencing something else? I suggest to rephrase anyway.

 12) "Mass production of these biofungicide bacteria formulations typically involves deep-tank liquid fermentation. However, in some cases, methods like semisolid or solid-state fermentation might be more suitable."

 Please explain, summarizing what these processes are and detailing why one could be preferred in certain conditions.

 13) Please explain what are the biopesticide properties of Agrobacterium tumefaciens and give recent detailed references for specific sentences.

 14) " *Agrobactenum tumefaciens* is available commercially in Australia, the United States, and New Zealand"

 What about other countries?

 Please also give examples of biopesticide commercial products containing A. tumefaciens.

 15) I also recommend the possibility to mention Agrobacterium radiobacter and how it is sometimes confused.

 16) "Entomopathogenic fungi are a type of fungi that can target and control pests without harming beneficial organisms such as pollinators. "

 This is not necessarily true and there is still the need to verify the safety of formulations against pollinators. For example: Ref (4)

 17) Abstract: "Biopesticides are pest control products derived from natural sources such as microbes, macro-organisms (insects and pathogens), plant extracts, and certain minerals."

 Reprised in the conclusions: " Biopesticides, derived from natural materials like animals, plants, bacteria, and certain minerals"

 This is a direct copy and paste from the EPA definition, which is credited in other sections: https://www.epa.gov/ingredients-used-pesticide-products/what-are-biopesticides

 I recommend to avoid such exact transpositions.

 18) The manuscript shifts in detail. Even within the same section, some parts are more accurate while others are more superficial. Examples are not always fully comprehensive. There are parts which are not fully clarified, and sometimes a specific example could induce to think that it is broadly applicable to other cases. I recommend to rework in order to convey the information in a clearer way.

 19) The article aims to explain recent advances, but it is for the most part an introductory presentation and summary of the known concepts in the field. I suggest to mention more in detail recent publications to discuss specifically what they add to the established literature. One example: Baculoviruses are introduced with references going back to the 90s, did they have recent innovations?

 20) The review mentions CRISPR/Cas9 as a tool for the development of microbial biopesticides. The brief mention doesn't explain why. I suggest to slightly expand.

 21) There are images of low quality, for example figure 8. Please create new images of higher quality.

 21) Do the photos of figure 6 have authors and copyright?

Is the review written in accessible language?

Partly

Are all factual statements correct and adequately supported by citations?

Partly

Are the conclusions drawn appropriate in the context of the current research literature?

Partly

Is the topic of the review discussed comprehensively in the context of the current literature?

Partly

Reviewer Expertise:

Environmental microbiology

I confirm that I have read this submission and believe that I have an appropriate level of expertise to confirm that it is of an acceptable scientific standard, however I have significant reservations, as outlined above.

## References

[1] : Recent paradigm shifts in the perception of the role of Bacillus thuringiensis in foodborne disease. *Food Microbiol* .2022;105: 10.1016/j.fm.2022.104025 104025 10.1016/j.fm.2022.104025 35473978

[2] : Insect Pathogenic Bacteria in Integrated Pest Management. *Insects* .2015;6(2) : 10.3390/insects6020352 352-67 10.3390/insects6020352 26463190 PMC4553484

[3] : Biopesticides and insect pollinators: Detrimental effects, outdated guidelines, and future directions. *Sci Total Environ* .2022;837: 10.1016/j.scitotenv.2022.155714 155714 10.1016/j.scitotenv.2022.155714 35525339

[4] : Laboratory Risk Assessment of Three Entomopathogenic Fungi Used for Pest Control toward Social Bee Pollinators. *Microorganisms* .2022;10(9) : 10.3390/microorganisms10091800 10.3390/microorganisms10091800 36144402 PMC9501116

# Response

**Published:** 2025-01-23

**Authors:** Kahsay Tadesse Mawcha

**Affiliations:** Bipesticides, International Centre for Genetic Engineering and Biotechnology, Cape Town, Western Cape, South Africa

Dear Reviewer,

 We appreciate your time and appreciate your comments and suggestions. We will address them in the main text of the manuscript.

# Response

**Published:** 2025-01-23

**Authors:** Kahsay Tadesse Mawcha

**Affiliations:** Bipesticides, International Centre for Genetic Engineering and Biotechnology, Cape Town, Western Cape, South Africa

**Authors' response to reviewers' comments**

**We thank the reviewer for their time and expertise in evaluating our review paper.**

 Thanks to the authors for writing a long review about this topic. I have some minor corrections and suggestions about the manuscript. Please forgive me in advance if my language is not clear enough or heavy to read:

 1. The review in general could benefit from a more synthetic language. Often, paragraphs can be shortened while conveying the same amount of information. Occasionally, it also happens that the same concepts are repeated, for example when illustrating the several advantages of biopesticides or how they align with several different target strategies. There are other examples that could benefit from reading again the text and summarizing repetitions. I suggest the authors to consider this possibility, as it can help to promote their ideas in a more effective and concise way. 

  

- **Authors Response**: Thank you for your comment, and we made a few changes to the text.

2. "However, resistance to some *Bt* products has been noted.". I recommend adding a brief mention of how and why this might have happened.

  

- **Response**: Thank you for your question. For the “How?” through natural selection. Insect pests that are susceptible to Bt toxins die, and those resistant can survive and reproduce.

3. It could be worthy to note that recently some concerns have arisen about the possibility that Bt toxins could be involved in a fraction of foodborne outbreaks, although there are still relevant gaps in knowledge that needs to be filled and we cannot yet draw definitive conclusions. Still, the reader might be interested in pursuing further studying and investigation of the topic. A reference, Ref (1)

 Not strictly necessary, depending on the goals.

  

- **Response**: Implementing several strategies, including cultivating refuge areas of non-Bt crops, alternating Bt crops with non-Bt varieties, and employing different Bt toxins that operate via various mechanisms, may reduce the threat of resistance. However, the effectiveness of these strategies can vary depending on the specific insect species and the environmental conditions.

 

 4. There are other entomopathogenic bacteria of interest such as Lysinibacillus sphaericus, Brevibacillus laterosporus, Serratia marcescens, Yersinia entomophaga etc.. The topic is much larger and has quite interesting considerations regarding the feasibility of applications, their effectiveness, and the large-scale limitations. For example: Ref (2)

  

- **Response: Thanks for your comments**. We have included the above bacterial biopesticides in the main text.  Other entomopathogenic bacteria of interest include *Chromobacterium subtsugae, Brevibacillus laterosporus, Lysinibacillus sphaericus, Paenibacillus popilliae, Serratia marcescens, *and *Yersinia entomophagy *(Gangwar et al. 2021).

Gangwar, P., Trivedi, M., Tiwari, R.K. (2021). Entomopathogenic Bacteria. In: Omkar (eds) Microbial Approaches for Insect Pest Management. Springer, Singapore. https://doi.org/10.1007/978-981-16-3595-3_2

 5) "Besides, biopesticides 1) generate fewer greenhouse gasses (GHG) than chemical pesticides, 2) improve nutrient availability for plants, 3) increase soil fertility, and 4) offer a more environmentally safe approach to pest control in agriculture, which can be useful in the fight against climate change."

 Please provide references for these points.

- **Response**: We have included several references from FAO 2017; 2020; Climate-smart, and the Paris Agreement. We have also added Fenibo et al. (2022)

Fenibo EO, Ijoma GN, Nurmahomed W, Matambo T. The Potential and Green Chemistry Attributes of Biopesticides for Sustainable Agriculture. *Sustainability*. 2022; 14(21):14417. https://doi.org/10.3390/su142114417

 5) Please elaborate points 2 and 3: if you are stating that biopesticides per se are also biostimulants, this requires a better explanation.

 Please expand point 4 or condensate it with point 1.

- **Response**: Thank you for your suggestion. However, the two points are clear, and we believe there is no need to elaborate.

6) "Microbial biopesticides offer diverse delivery methods to reach crop targets effectively. These methods include live organisms, dead organisms, and spores"

 Please describe these methods here for a better understanding of the scientific mechanisms.

- **Response**: Thank you for your suggestion. We have addressed and incorporated your comment.

7) "Additionally, they leave no harmful residues, don’t harm pollinators and other valuable organisms, and can be applied close to harvest time."

 This is actually a slight approximation. Recent research show that there could be some negative effects after all, and that there are knowledge gaps regarding pollinators. It is always a matter of balancing risks and benefits. While the low impact status of biopesticides is not changed, it is still worth to remember peculiarities and potential limits. For reference: Ref (3)

  

- **Response**: Many Thanks for your suggestion

8) "Bacterial biopesticides, while effective, come with a few disadvantages. For instance, they only target specific species or groups of insects, leaving other pests to survive and continue causing damage. "

 This is conceptually a bit clashing with the previous statement that specificity is an advantage, in order to avoid non-target insects. It is better to reformulate in a clearer way that the specificity gives that advantages but requires complementarity of treatment when dealing with different pests.

- **Response**: Thank you for your suggestions and comments. We have addressed them in the main text of the manuscript.

9) The review doesn't adhere to the same structure within its sections. For example, section 5.5 gives two explicit separated paragraphs titled "advantages" and "disadvantages" of EPNs, while other sections discuss them as part of the flow in the text. I suggest to uniform the style for better clarity and readibility.

- **Response**: Thank you for your suggestions and comments. We have mentioned the advantages and disadvantages of the other types of microbial pesticides. We did this purposefully, as they are different in type.

10) Section 6 is a bit superficial and doesn't mention the need to assess that alloctonous predator can be safely controlled - i.e. they cannot become invasive. Since the review focuses on microbials, it doesn't need to go in detail about macrobials, but better accuracy is required.

  

- **Response**: Thank you for your suggestions. Macrobial agents (living organisms, such as insects, mites, or nematodes, that are used to control pests) and microbial agents (microorganisms, such as bacteria, fungi, or viruses, that are used to control pests) are both used in pest control. Still, they differ significantly in their nature and mode of action. While both macrobial and microbial agents are biological control methods, they differ in their composition and how they target pests. Macrobial agents are living organisms that directly attack the pest, whereas microbial agents are microorganisms that can infect, kill, or produce toxins harmful to the pest. So, the focus of this review is to discuss microbial biopesticides. 

11) Some phrases like "reduce chomping on your precious plants", "This gram-positive warrior wields a powerful weapon: delta-endotoxin proteins that wreak havoc on the mid-guts of susceptible insect larvae" or "These technologies offer exciting possibilities for sustainable agriculture" sound quite odd and out of place for a technical review. Are they referencing something else? I suggest to rephrase anyway.

- **Response**: Thank you for your suggestions. We have rephrased it in the main text of the manuscript as “Meenatchi and Aditi (2021) and Gautam (2008) have summarized several promising biopesticides that are effective against various crop pests. These specialized assassins are laser-guided for specific pests, efficiently hunting them even at low levels. They curb reproduction, keep infestations below damage thresholds, and play well with other pest-management tactics. Additionally, they help disrupt pest feeding and lessen the damage to your plants.  They are environmentally safe pest control solutions “

12) "Mass production of these biofungicide bacteria formulations typically involves deep-tank liquid fermentation. However, in some cases, methods like semisolid or solid-state fermentation might be more suitable."

 Please explain, summarizing what these processes are and detailing why one could be preferred in certain conditions.

- **Response**: Thank you for your suggestions.The mass production of biofungicide bacteria formulations typically involves deep-tank liquid fermentation for several reasons: high yield, controlled environment, scalability, automation and Cost-effectiveness.  If we explain it more than the present, it may need more pages, and this could be beyond the page limit of the review.

13) Please explain what are the biopesticide properties of Agrobacterium tumefaciens and give recent detailed references for specific sentences.

**Response**: Thank you for your suggestions. We have included the following references.

  

- Abdallah, Dorra Ben, Slim Tounsi, Houda Gharsallah, Adnane Hammami, and Olfa Frikha-Gargouri. "Lipopeptides from Bacillus amyloliquefaciens strain 32a as promising biocontrol compounds against the plant pathogen Agrobacterium tumefaciens." *Environmental Science and Pollution Research* 25 (2018): 36518-36529.
- Brown, Pamela JB, Jeff H. Chang, and Clay Fuqua. "Agrobacterium tumefaciens: a transformative agent for fundamental insights into host-microbe interactions, genome biology, chemical signaling, and cell biology." *Journal of Bacteriology* 205, no. 4 (2023): e00005-23.
- Mnif, Inès, and Dhouha Ghribi. "Potential of bacterial derived biopesticides in pest management." *Crop Protection* 77 (2015): 52-64.

14) " *Agrobactenum tumefaciens* is available commercially in Australia, the United States, and New Zealand"

 What about other countries?

 Please also give examples of commercial biopesticide products containing A. tumefaciens.

- **Response**: Thank you for your suggestions There are no recent *A. tumefaciens* products 

15) I also recommend the possibility to mention Agrobacterium radiobacter and how it is sometimes confused.

- **Response**: Thank you for your suggestions and comments. A strain of *A. radiobacter* known as K84 is used as a biopesticide to control crown gall disease (Stockwell et al. 1993).

Stockwell, V. O., L. W. Moore, and J. E. Loper. "Fate of Agrobacterium radiobacter K84 in the environment." *Applied and Environmental Microbiology* 59, no. 7 (1993): 2112-2120.

 16) "Entomopathogenic fungi are a type of fungi that can target and control pests without harming beneficial organisms such as pollinators. "

 This is not necessarily true and there is still the need to verify the safety of formulations against pollinators. For example: Ref (4)

- **Response: Thank you for your suggestions and comments**. The statement “Entomopathogenic fungi are a type of fungi that can target and control pests without harming beneficial organisms” is clear. Without harming beneficial organisms, such organisms cause minimal or no damage to beneficial organisms. 

17) Abstract: "Biopesticides are pest control products derived from natural sources such as microbes, macro-organisms (insects and pathogens), plant extracts, and certain minerals."

 Reprised in the conclusions: " Biopesticides, derived from natural materials like animals, plants, bacteria, and certain minerals"

 This is a direct copy and paste from the EPA definition, which is credited in other sections: https://www.epa.gov/ingredients-used-pesticide-products/what-are-biopesticides

 I recommend to avoid such exact transpositions.

- **Response: Thank you for your suggestions and comments. **We have addressed the comment in the main text of the manuscript **“**Biopesticides consist of naturally occurring substances, including insects, nematodes, microorganisms, plants, and semiochemicals, along with their by-products. They offer a sustainable alternative to traditional chemical pesticides.

18) The manuscript shifts in detail. Even within the same section, some parts are more accurate while others are more superficial. Examples are not always fully comprehensive. There are parts which are not fully clarified, and sometimes a specific example could induce to think that it is broadly applicable to other cases. I recommend to rework in order to convey the information in a clearer way.

- **Response: Thank you for your suggestions and comments, and we have made changes to the main text. However, this comment is very general one.**

19) The article aims to explain recent advances, but it is for the most part an introductory presentation and summary of the known concepts in the field. I suggest to mention more in detail recent publications to discuss specifically what they add to the established literature. One example: Baculoviruses are introduced with references going back to the 90s, did they have recent innovations?

**Response: Thank you for your suggestions and comments. We have added the following recent references to the list.**

- Das, Sumistha, Arunava Goswami, and Nitai Debnath. "Application of baculoviruses as biopesticides and the possibilities of nanoparticle mediated delivery." In *Nano-biopesticides today and future perspectives*, pp. 261-280. Academic Press, 2019.
- Gelaye, Yohannes, and Belete Negash. "The role of baculoviruses in controlling insect pests: A review." *Cogent Food & Agriculture* 9, no. 1 (2023): 2254139.
- Ruiu, Luca. "Microbial biopesticides in agroecosystems." *Agronomy* 8, no. 11 (2018): 235.
- Wilson, Kenneth, David Grzywacz, Igor Curcic, Freya Scoates, Karen Harper, Annabel Rice, Nigel Paul, and Aoife Dillon. "A novel formulation technology for baculoviruses protects biopesticide from degradation by ultraviolet radiation." Scientific Reports 10, no. 1 (2020): 13301.

20) The review mentions CRISPR/Cas9 as a tool for the development of microbial biopesticides. The brief mention doesn't explain why. I suggest to slightly expand.

- **Response: Thank you for your suggestions and comments**. However, the current content of the paragraph about CRISPR/Cas9 is sufficient, and we believe that adding more information on CRISPR/Cas9 may become overwhelming.

21) There are images of low quality, for example figure 8. Please create new images of higher quality.

- **Response: Thank you for your suggestions and comments**

21) Do the photos of figure 6 have authors and copyright?

- **Response: Thank you for your suggestions and comments.** **Yes. It is our own work.**

# Reviewer report: Reviewer response for version 3

**DOI:** 10.5256/f1000research.174749.r348089

**Published:** 2024-12-30

**Authors:** Sonia Sethi

**Affiliations:** 1 Dr. B. Lal Institute of Biotechnology, Malviya Nagar, Jaipur, India

Written properly 

 Recent advances in biopesticide research highlight microbial agents like bacteria, fungi, and viruses as eco-friendly alternatives to chemical pesticides. Innovations focus on enhancing efficacy, formulation stability, and scalability for agricultural use. Genetic engineering, nanotechnology, and integrated pest management strategies are driving improvements, addressing resistance issues, and promoting sustainable crop protection globally.

Is the review written in accessible language?

Yes

Are all factual statements correct and adequately supported by citations?

Yes

Are the conclusions drawn appropriate in the context of the current research literature?

Yes

Is the topic of the review discussed comprehensively in the context of the current literature?

Yes

Reviewer Expertise:

Agriculture microbiology

I confirm that I have read this submission and believe that I have an appropriate level of expertise to confirm that it is of an acceptable scientific standard.

# Reviewer report: Reviewer response for version 3

**DOI:** 10.5256/f1000research.174749.r346594

**Published:** 2024-12-27

**Authors:** Mehari Desta Hawku

**Affiliations:** 1 Crop Sciences, University of Rwanda College of Agriculture Animal Sciences and Veterinary Medicine (Ringgold ID: 183043), Busogo, Musanze, Rwanda

No further comment.

Is the review written in accessible language?

Yes

Are all factual statements correct and adequately supported by citations?

Yes

Are the conclusions drawn appropriate in the context of the current research literature?

Yes

Is the topic of the review discussed comprehensively in the context of the current literature?

Yes

Reviewer Expertise:

My research interests encompass a broad spectrum of plant pathology including:Identification and molecular characterization of resistance or susceptible genes in plant-microbe interactions;Understanding the intricate mechanisms governinginteractions between plants and microbes;Examining the signaling pathways involved in activating plant defense responses against pathogens;Studying the molecular components that enable pathogens to invade and manipulate plant cells;Understanding the mechanisms of abiotic stress tolerance in plants

I confirm that I have read this submission and believe that I have an appropriate level of expertise to confirm that it is of an acceptable scientific standard.

# Reviewer report: Reviewer response for version 2

**DOI:** 10.5256/f1000research.173837.r335979

**Published:** 2024-11-06

**Authors:** Yordanys Ramos

**Affiliations:** 1 Instituto de Investigaciones Agropecuarias y Forestales, Universidad Michoacana de San Nicolas de Hidalgo, Morelia, Michoacán, Mexico

The authors have made the relevant modifications to the manuscript and have explained in detail certain aspects that helped improve the understanding of the paper.

Is the review written in accessible language?

Yes

Are all factual statements correct and adequately supported by citations?

Yes

Are the conclusions drawn appropriate in the context of the current research literature?

Yes

Is the topic of the review discussed comprehensively in the context of the current literature?

Partly

Reviewer Expertise:

Biological Control, Integrated Pest Management, Entomology, Microbiology

I confirm that I have read this submission and believe that I have an appropriate level of expertise to confirm that it is of an acceptable scientific standard.

# Reviewer report: Reviewer response for version 2

**DOI:** 10.5256/f1000research.173837.r335978

**Published:** 2024-11-04

**Authors:** Mehari Desta Hawku

**Affiliations:** 1 Crop Sciences, University of Rwanda College of Agriculture Animal Sciences and Veterinary Medicine (Ringgold ID: 183043), Busogo, Musanze, Rwanda

No further comments.

Is the review written in accessible language?

Yes

Are all factual statements correct and adequately supported by citations?

Yes

Are the conclusions drawn appropriate in the context of the current research literature?

Yes

Is the topic of the review discussed comprehensively in the context of the current literature?

Yes

Reviewer Expertise:

My research interests encompass a broad spectrum of plant pathology including:Identification and molecular characterization of resistance or susceptible genes in plant-microbe interactions;Understanding the intricate mechanisms governinginteractions between plants and microbes;Examining the signaling pathways involved in activating plant defense responses against pathogens;Studying the molecular components that enable pathogens to invade and manipulate plant cells;Understanding the mechanisms of abiotic stress tolerance in plants

I confirm that I have read this submission and believe that I have an appropriate level of expertise to confirm that it is of an acceptable scientific standard.

# Response

**Published:** 2024-11-04

**Authors:** Kahsay Tadesse Mawcha

**Affiliations:** Bipesticides, International Centre for Genetic Engineering and Biotechnology, Cape Town, Western Cape, South Africa

We really appreciate your time and expertise. Many thanks for approving our article.

# Reviewer report: Reviewer response for version 1

**DOI:** 10.5256/f1000research.169409.r326009

**Published:** 2024-10-16

**Authors:** Mehari Desta Hawku

**Affiliations:** 1 Crop Sciences, University of Rwanda College of Agriculture Animal Sciences and Veterinary Medicine (Ringgold ID: 183043), Busogo, Musanze, Rwanda

The manuscript investigates the rising significance of microbial biopesticides as eco-friendly alternatives to traditional pesticides. It emphasizes the potential of utilizing natural organisms, such as bacteria, fungi, viruses, nematodes and protozoans, for effective pest control while reducing environmental harm. The authors provide a thorough examination of recent advancements and methodologies that enhance the effectiveness of these biopesticides. Furthermore, the review underscores the importance of integrating these biopesticides into comprehensive integrated pest management strategies. However, to offer a more comprehensive review, the following aspects should be addressed:

1. While the topic is broad, the literature focuses only on the role of microbial biopesticides in controlling insect pests, overlooking their role in disease and weed control
2. The role of recent cutting-edge technologies, such as CRISPR/Cas9 and RNA interference (RNAi), was not thoroughly discussed.
3. Regulatory frameworks and commercialization aspects, were not addressed.

**Minor comments:**

**Section 1: Introduction**

1. The introduction section lacks a comprehensive summary that clearly informs readers about the review's aims, the specific points the literature addresses, as well as the knowledge gaps and suggested future research directions. It is essential to include a concise overview outlining the main themes covered in the review. Additionally, highlighting the role of emerging technologies, such as CRISPR/Cas9 and RNA interference (RNAi) in biopesticide development, along with the potential for integrating biopesticides into sustainable agricultural practices, will provide clearer context for the reader. This summary should also emphasize the importance of interdisciplinary collaboration among researchers, policymakers, practitioners, and manufacturers to enhance the efficacy and acceptance of biopesticides in agricultural systems.
2. The image in Figure 1 is blurry; please consider replacing it with a clearer image and remove the word "legends". From the description of Figure 1, it appears that you have missed one image. Therefore, you can either remove the sentence, "The second part demonstrates how the five steps for implementing CSA intersect with and affect the 17 SDGs in terms of synergies and trade-offs," or consider incorporating the missing image.

**Section 2:** **Methods**

 The methodology section is missing some key points, as outlined below: 

1. It is important to clarify the specific research questions guiding the review  
2. It’s unclear how studies were selected for review. Please include specific inclusion and exclusion criteria used for selecting papers
3. The method does not specify the keywords or phrases used during the search process.
4. There’s no description of how potential biases in selecting literature were mitigated, nor is there any indication of measures taken to avoid confirmation bias.
5. Mentioning how the quality of the papers was assessed would add rigor to the methodology
6. It would be helpful to specify the types of studies reviewed or considered for inclusion
7. Please specify the time frame of the literature reviewed
8. If there was any geographical focus, it would be beneficial to mention it.

**Section** **3** **:** **Concept and classification of biopesticides**

1. The second paragraph in this section is not related to the topic. Instead, this concept should be discussed under section 4.
2. The idea in the third paragraph should also be addressed on a separate topic as Mechanism of action and efficacy of microbial biopesticides

**Section** **4** **:** **Importance of biopesticides in sustainable** **agriculture**

1. The sentence ‘The use of  biopesticides has steadily increased by a steady 11.0% Compound Annual Growth Rate (CAGR) between 2018 and 2022.’ under section 4 should be rephrased as ‘The use of biopesticides has steadily increased at a Compound Annual Growth Rate (CAGR) of 11.0% between 2018 and 2022.’ to avoid the redundancy of the word ‘steadily’ and ‘steady’
2. Under this section the following points should be discussed in details; Environmental Safety, Reduced Chemical Dependency, Promoting Biodiversity, Compatibility with Organic Farming, and Sustainability and Resilience .

**Section 5:** **Role of biopesticides in integrated pest management**

1. The different microbial biopesticides should be discussed under subsection 5.1 as a sub-subsection not as a subsection.
2. In table 1, the role of microbial biopesticides in the IPM program is not mentioned within the table; therefore, please consider modifying the table's title.
3. In Table 1, I suggest adding a fifth column to list the references 
4. In Table 1, could you clarify what is meant by 'application type'? If it refers to the method of delivery, this is not clearly specified. In addition, the mode of action is missing in several instances under the column ‘Application Type and Mode of Action’. For some microbial biopesticides, important details are absent within the table. On Page 9, under Table 1, please replace 'fungal-based biopesticides' with 'viral-based biopesticides.' Overall, Table 1 lacks consistency and would benefit from clearer definitions and more complete information.

**Subsection 5.2:** **Bacterial based biopesticides**

1. After using the full name *Bacillus thuringiensis* (Bt), you should continue using its abbreviated form (Bt) throughout the text.
2. The last paragraph under sub-section 5.2 should be supported by relevant literature.
3. The image (particularly the text) in Figure 2 is unclear; please consider replacing it with a clearer version. Additionally, remove the word 'legend' as well as the phrase 'The mode of action of Bt gene and Cry protein,' since this information is already specified in the figure itself

**Subsection 5.3:  Fungal biopesticides**

 A citation is needed for Figure 3. Such figures are typically self-explanatory and do not require legends, so please remove the note stating, ‘Legends are indicated in the figures. No additional legends.’

**Subsection 5.4: Viral biopesticides**

1. Please replace ‘Baculovirus family’ with *Baculoviridae* **and do not italicize baculovirus
2. Would you please reconsider the first sentence of the third paragraph in subsection 5.4 to maintain clarity?
3. In Figure 4 (Page 12), please remove the term 'legends,' as it is unnecessary to mention since it is evident that you are describing the figure. Additionally, please ensure you provide proper citations for this figure, as you are referencing two sources: Singh (2019) and Kalamkoff (2004).  
4. On page 13, the same legend is repeated; please consider removing it.

**Subsection 5.5: Nematodes as biopesticides**

1. Please add a citation after the caption of Figure 5 and remove the citation from the description. Figure 5 is somewhat blurry; please use a clearer image and consider removing the word ‘legends’.

In **subsection 5.6**, please consider not italicizing the word ‘septicaemia’

**Section 6: Natural enemies of pests as biopesticides**

1. Since the primary focus of this review is on microbial biopesticides, and predators and parasitoids are classified as macrobials, I suggest removing this section.
2. The image (particularly the text) in Figure 6 is not clear; please consider replacing it.
3. Please add a citation to Figure 6.

Please add a citation to Figure 7 and consider using a clear image

**Subsection 8.2:** **Factors affecting efficacy of biopesticides**

1. Under this subsection, the first sentence should be modified as ‘Various challenges and limitations, including environmental conditions such as temperature, humidity, and sunlight, can influence the efficacy of biopesticides during pest control’
2. In the second paragraph, please check the citation related to ‘ (International Symposium on Food Safety and Control).’
3. The image in Figure 8 is blurry; please consider using a clearer and more visually appealing image. I recommend using photo editing software, such as Photoshop, to enhance the quality. Additionally, please remove the phrase 'own work' from the caption. Finally, ensure that the figure is either self-explanatory or includes a legend where necessary.

**Section 9.** **Future perspectives and research directions**

 Please remove subsection 9.1 from this section, as there are no other subsections following it. Additionally, avoid using a single sentence as a paragraph; instead, merge it with the preceding paragraph. In this section on future perspectives and research directions, it would be valuable to discuss in more detail the role of the cutting-edge technologies, such as CRISPR/Cas9 and RNA interference (RNAi) in pest control.

**Section 10. Conclusion**

 The conclusion effectively encapsulates the key findings regarding the benefits and challenges of biopesticides. However, the sentence stating that "novel microbial strain creation with improved features, such as greater efficacy and larger target spectra, should be prioritized in future research and the use of biopesticides" may be more appropriate in a future perspectives section. This sentence emphasizes the need for ongoing research and innovation, which aligns better with a section dedicated to future research directions.

Is the review written in accessible language?

Yes

Are all factual statements correct and adequately supported by citations?

Yes

Are the conclusions drawn appropriate in the context of the current research literature?

Yes

Is the topic of the review discussed comprehensively in the context of the current literature?

Yes

Reviewer Expertise:

My research interests encompass a broad spectrum of plant pathology including:Identification and molecular characterization of resistance or susceptible genes in plant-microbe interactions;Understanding the intricate mechanisms governinginteractions between plants and microbes;Examining the signaling pathways involved in activating plant defense responses against pathogens;Studying the molecular components that enable pathogens to invade and manipulate plant cells;Understanding the mechanisms of abiotic stress tolerance in plants

I confirm that I have read this submission and believe that I have an appropriate level of expertise to confirm that it is of an acceptable scientific standard, however I have significant reservations, as outlined above.

# Response

**Published:** 2024-10-21

**Authors:** Kahsay Tadesse Mawcha

**Affiliations:** Bipesticides, International Centre for Genetic Engineering and Biotechnology, Cape Town, Western Cape, South Africa

Dear Chief Editor,

 Dear Reviewer,

 We have carefully considered all of the reviewers' comments and suggestions. To address these, we have made substantial revisions to the manuscript. Below, we provide a detailed response to each specific point raised.

 We believe that these revisions have significantly strengthened the manuscript and improved its clarity, comprehensiveness, and overall quality. We are confident that the revised manuscript now meets the high standards of your journal.

 Thank you again for your valuable feedback.

 Sincerely,

**Reviewer 2**

**Comments and responses**

 The manuscript investigates the rising significance of microbial biopesticides as eco-friendly alternatives to traditional pesticides. It emphasizes the potential of utilizing natural organisms, such as bacteria, fungi, viruses, nematodes and protozoans, for effective pest control while reducing environmental harm. The authors provide a thorough examination of recent advancements and methodologies that enhance the effectiveness of these biopesticides. Furthermore, the review underscores the importance of integrating these biopesticides into comprehensive integrated pest management strategies. However, to offer a more comprehensive review, the following aspects should be addressed:

1. While the topic is broad, the literature focuses only on the role of microbial biopesticides in controlling insect pests, overlooking their role in disease and weed control

- We accept the reviewer’s comment. To address his comment, we have modified the title of the review to focus on Microbial biopesticides and recent advancements and methodologies

1. The role of recent cutting-edge technologies, such as CRISPR/Cas9 and RNA interference (RNAi), was not thoroughly discussed

- As the focus of the current review is microbial biopesticides, CRISPR/Cas9 and RNA interference (RNAi) have been discussed in detail under the emerging trends in biopesticides. We believe that there is no need to discuss them as a subheading.

1. Regulatory frameworks and commercialization aspects were not addressed.

- The review focuses on summarizing previous and recent studies about the effectiveness of biopesticides, particularly microbial products. Hence, regulatory frameworks and commercialization aspects are beyond the scope of the review. However, we have discussed a few points under the conclusion part about the importance of Regulatory frameworks and the need for harmonization of biopesticide guidelines as a future work.

We have also modified the title “ **Recent Advances in Biopesticide Research and Development with A Focus on Microbials**”

**Minor comments:**

**Section 1: Introduction**

1. The introduction section lacks a comprehensive summary that clearly informs readers about the review's aims, the specific points the literature addresses, as well as the knowledge gaps and suggested future research directions. It is essential to include a concise overview outlining the main themes covered in the review. Additionally, highlighting the role of emerging technologies, such as CRISPR/Cas9 and RNA interference (RNAi) in **biopesticide development, along with the potential for integrating biopesticides into sustainable agricultural practices, ** will provide a clearer context for the reader. This summary should also emphasize the importance of interdisciplinary collaboration among researchers, policymakers, practitioners, and manufacturers to enhance the efficacy and acceptance of biopesticides in agricultural systems.

- We appreciate the suggestions given by the reviewer. However, most of the comments are out of the scope of the review content. Some of the comments, such as the role of emerging technologies, such as CRISPR/Cas9 and RNA interference (RNAi), and the potential for integrating biopesticides into sustainable agricultural practices, are highlighted in the conclusion part.

1. The image in Figure 1 is blurry; please consider replacing it with a clearer image and remove the word "legends". From the description of Figure 1, it appears that you have missed one image. Therefore, you can either remove the sentence, "The second part demonstrates how the five steps for implementing CSA intersect with and affect the 17 SDGs in terms of synergies and trade-offs," or consider incorporating the missing image.

- Within the figure, sections show the different linkages with climate-smart agriculture and how they are linked to the 17 SDGs. Hence, there is no missed image in this section. The figure provides information that is interlinked with each other. Climate-smart agriculture (CSA) has a strong potential to contribute to the achievement of the 17 Sustainable Development Goals (SDGs). A critical precondition for realising this scope is identifying potential synergies and trade-offs between the 3 pillars of CSA and the 5 implementation steps of CSA and the SDGs. These provide entry points for targeted CSA planning so as to enhance synergies and reduce trade-offs. CSA and the SDGs have synergies and trade-offs at different levels for each of the 3 pillars of CSA where some of the synergies & trade-offs overlap all 3 pillars of CSA for some of the SDGs, namely SDGs 2 and 6.  The overlapping synergies & trade-offs for the implementation steps and SDGs are different, as only step 2 has overlapping trade-offs and synergies in SDG’s, 6,9,13, and 15.
- We have also improved the paragraph and made some changes to it.

**Section 2: Methods**

 The methodology section is missing some key points, as outlined below: 

1. It is important to clarify the specific research questions guiding the review  

- What are the current trends and advancements in the development and application of microbial biopesticides?
- What are the key challenges and limitations associated with the use of microbial biopesticides in agricultural settings?
- What are the most promising microbial agents currently being developed for use as biopesticides? Are the most important guiding questions

1. It’s unclear how studies were selected for review. Please include specific inclusion and exclusion criteria used for selecting papers

- Only microbial (bacterial, fungal, viral, nematode, and protozoa) biopesticides were included and other types were excluded

1. The method does not specify the keywords or phrases used during the search process.

- Using various keywords, including *biocontrol*, formulations, bioprotection, climate-smart agriculture, and sustainable pest management, 197 relevant studies were found. We have already included the following phrases: The literature outcomes were categorised into the (i) classification of biopesticides, (ii) importance of biopesticides in sustainable agriculture, (iii) role of microbial biopesticides in integrated pest management, (iv) different types and formulations of microbial biopesticides, and (v) advancement and future perspectives of microbial biopesticides.

1. There’s no description of how potential biases in selecting literature were mitigated, nor is there any indication of measures taken to avoid confirmation bias.

- To avoid biases in selecting literature, we used articles that are published in peer-reviewed journals, which means available on the following search engines, Google Scholar, PubMed, Scopus (Elsevier), Web of Science, Semantic Scholar, Academia

1. Mentioning how the quality of the papers was assessed would add rigor to the methodology

- Most of our sources are published in peer-reviewed journals.

1. It would be helpful to specify the types of studies reviewed or considered for inclusion

- We have included original research articles and reviews published in peer-reviewed journals and other relevant resources

1. Please specify the time frame of the literature reviewed

- Mostly, we used recently published articles (2018-2024) and very few; we have also included > 5 references, which are from 2003>.

1. If there were any geographical focus, it would be beneficial to mention it.

- The review is an overview of the current research and developments in microbial biopesticides. So, there are no specific geographic limitations.

**Section 3: Concept and classification of biopesticides**

1. The second paragraph in this section is unrelated to the topic. Instead, this concept should be discussed under section 4.

- Section 3 deals with the classification/definition of biopesticides (general) by various organizations and associations. So, it is better to be as it is.

1. The idea in the third paragraph should also be addressed on a separate topic as Mechanism of action and efficacy of microbial biopesticides

- We accept the suggestion however, as we have discussed the mode of action and efficacy of each microbial product, this subtitle may cause a repetition.

**Section 4: Importance of biopesticides in sustainable agriculture**

1. The sentence ‘The use of biopesticides has steadily increased by a steady 11.0% Compound Annual Growth Rate (CAGR) between 2018 and 2022.’ under section 4 should be rephrased as ‘The use of biopesticides has steadily increased at a Compound Annual Growth Rate (CAGR) of 11.0% between 2018 and 2022.’ to avoid the redundancy of the word ‘steadily’ and ‘steady’

- Accepted and addressed.

1. Under this section, the following points should be discussed in detail: Environmental Safety, Reduced Chemical Dependency, Promoting Biodiversity, Compatibility with Organic Farming, and Sustainability and Resilience.

- We are happy to discuss them in detail however, we may go beyond the scope of the topic!

**Section 5: Role of biopesticides in integrated pest management**

1. The different microbial biopesticides should be discussed under subsection 5.1 as a sub-subsection, not as a subsection.

- Comment Accepted. However, we discuss the various types of biopesticides, including Biochemical, Botanical, and pheromone-based; the world limit and the number of pages may exceed the limit. So, it is better to focus on the title-i.e. microbial-based biopesticides!

1. In Table 1, the role of microbial biopesticides in the IPM program is not mentioned within the table; therefore, please consider modifying the table's title.

- Comment accepted, and Table 1. has been modified as “Microbial biopesticide categories, mode of action, and their use in pest management.

1. In Table 1, I suggest adding a fifth column to list the references 

- We have included the references in line with each column.

1. In Table 1, could you clarify what is meant by 'application type'? If it refers to the method of delivery, this is not clearly specified. In addition, the mode of action is missing in several instances under the column ‘Application Type and Mode of Action’. For some microbial biopesticides, important details are absent within the table.

- The table is self-explanatory and the title is also modified as suggested. The “'application type'” is NOT referring to the method. It is referring to “use”. The remark column is for additional information.

1. On Page 9, under Table 1, please replace 'fungal-based biopesticides' with 'viral-based biopesticides.' Overall, Table 1 lacks consistency and would benefit from clearer definitions and more complete information.

- Accepted, but as the title of the table is formatted by the journal editor, it will be corrected during the production.

**Subsection 5.2: Bacterial-based biopesticides**

1. After using the full name *Bacillus thuringiensis* (Bt), you should continue using its abbreviated form (Bt) throughout the text.

- Yes, but when we start a new line, we normally use the full name, that is, species and genus names.

1. The last paragraph under sub-section 5.2 should be supported by relevant literature.

- All paragraphs are cited. It is not clear!

1. The image (particularly the text) in Figure 2 is unclear; please consider replacing it with a clearer version. Additionally, remove the word 'legend' as well as the phrase 'The mode of action of Bt gene and Cry protein,' since this information is already specified in the figure itself

- Okay. Accepted, and the legend is deleted.

**Subsection 5.3:  Fungal biopesticides**

1. A citation is needed for Figure 3. Such figures are typically self-explanatory and do not require legends, so please remove the note stating, ‘Legends are indicated in the figures. No additional legends.’

Okay. Accepted. The figure is our own work, and there is no need to include citations.

**Subsection 5.4: Viral biopesticides**

1. Please replace ‘Baculovirus family’ with Baculoviridae **and do not italicise baculovirus

- Done!

1. Would you please reconsider the first sentence of the third paragraph in subsection 5.4 to maintain clarity?

- Accepted and modified as “Figure 4. shows how viral replication and infection take place and attack their target host”.

1. In Figure 4 (Page 12), please remove the term 'legends,' as it is unnecessary to mention since it is evident that you are describing the figure. Additionally, please ensure you provide proper citations for this figure, as you are referencing two sources: Singh (2019) and Kalamkoff (2004).  

- Accepted and the “Figure 4. Mode of action of viral biopesticides ( Singh et al. 2019).” It was deleted in the text.

1. On page 13, the same legend is repeated; please consider removing it.

- Okay. Done!

**Subsection 5.5: Nematodes as biopesticides**

1. Please add a citation after the caption of Figure 5 and remove the citation from the description. Figure 5 is somewhat blurry; please use a clearer image and consider removing the word ‘legends’.

- Accepted. We have deleted the term “legend”. However, the Figure is our own work, and there is no need to put citations.

1. In **subsection 5.6**, please consider not italicizing the word ‘septicaemia’

- Accepted and done!

**Section 6: Natural enemies of pests as biopesticides**

1. Since the primary focus of this review is on microbial biopesticides, and predators and parasitoids are classified as macrobials, I suggest removing this section.

- Sometimes, the definition of biopesticides may include parasitoids and predators, and their inclusion in this review enhances the broader reader of the journal.

1. The image (particularly the text) in Figure 6 is unclear; please consider replacing it.
2. Please add a citation to Figure 6.

- The figure is our own work and no need to include a citation.

1. Please add a citation to Figure 7 and consider using a clear image

- The figure is our own work and no need to include a citation.

**Subsection 8.2: Factors affecting efficacy of biopesticides**

1. Under this subsection, the first sentence should be modified as ‘Various challenges and limitations, including environmental conditions such as temperature, humidity, and sunlight, can influence the efficacy of biopesticides during pest control’

- Accepted and done!

1. In the second paragraph, please check the citation related to ‘ (International Symposium on Food Safety and Control).’

- Comment accepted. We have added the full information and hyperlinked the details.

https://conferences.iaea.org/event/351/

  

1. The image in Figure 8 is blurry; please consider using a clearer and more visually appealing image. I recommend using photo editing software, such as Photoshop, to enhance the quality. Additionally, please remove the phrase 'own work' from the caption. Finally, ensure that the figure is either self-explanatory or includes a legend where necessary.

- Accepted and done!

**Section 9. Future perspectives and research directions**

1.  Emerging trends in biopesticide development

Please remove subsection 9.1 from this section, as there are no other subsections following it. Additionally, avoid using a single sentence as a paragraph; instead, merge it with the preceding paragraph. In this section on future perspectives and research directions, it would be valuable to discuss in more detail the role of cutting-edge technologies, such as CRISPR/Cas9 and RNA interference (RNAi), in pest control.

- ***Accepted and addressed!***

By combining CRISPR/Cas9 and RNAi, researchers can develop microbial biopesticides with enhanced specificity, efficacy, and environmental safety.  These technologies offer exciting possibilities for the future of pest control and sustainable agriculture.

 Additionally, we have added “Future perspectives and research directions in the field of microbial biopesticides, including key research directions include Genetic engineering to improve microbial agents, synergistic interactions with other pest control methods, advanced formulation and delivery techniques, resistance management strategies, environmental impact assessments, integration into sustainable agriculture, and public awareness and adoption are essential for advancing their development and application. The future of microbial biopesticides looks promising, with ongoing research focusing on enhancing their efficacy, specificity, and environmental safety”.

**Section 10. Conclusion **

1. The conclusion effectively encapsulates the key findings regarding the benefits and challenges of biopesticides. However, the sentence stating that "novel microbial strain creation with improved features, such as greater efficacy and larger target spectra, should be prioritized in future research and the use of biopesticides" may be more appropriate in a future perspectives section. This sentence emphasizes the need for ongoing research and innovation, which aligns better with a section dedicated to future research directions.

- Yes but the conclusion part also needs to show the future perspectives in the area, so we keep this section as it is.

Many thanks for the time you took to improve the paper. 

 Sincerely,

 Kahsay Tadesse

# Reviewer report: Reviewer response for version 1

**DOI:** 10.5256/f1000research.169409.r326005

**Published:** 2024-10-04

**Authors:** Yordanys Ramos

**Affiliations:** 1 Instituto de Investigaciones Agropecuarias y Forestales, Universidad Michoacana de San Nicolas de Hidalgo, Morelia, Michoacán, Mexico

Introduction section, Page 3, last paragraph.Change *Beauveria bassiana* to *Beauveria spp.* since there are many more entomopathogenic species within the *Beauveria* genus, not just *Beauveria bassiana*. It would be more accurate to use the genus ( *Beauveria spp.*), just as was done with *Metarhizium*, *Isaria*, and *Verticillium* *spp*.

 In that same paragraph, the authors should change *Verticillium* to *Lecanicillium* according to the current classification of that fungal genus.

 Page 5. Concepts and classifications of biopesticides. The authors should provide a citation for the concept of biopesticide provided by SADC, just as they did for the concept provided by USEPA. Likewise, they should include a citation for the concept provided by The European Environmental Agency.

 Page 5: Please provide a citation for the Paris Agreement and the Nagoya Protocol. The authors can find both documents at these links:

https://unfccc.int/sites/default/files/english_paris_agreement.pdf

https://www.cbd.int/abs/doc/protocol/nagoya-protocol-en.pdf

 Page 6: Biopesticide Categories. The authors do not need to repeat the concept of biopesticide again, as this makes the review repetitive and less engaging for the reader, especially considering that a section was already dedicated to detailing the various existing concepts of this term.

 Page 11: Figure 3, Mode of Action of Entomopathogenic Fungi. The cycle refers to the infection of *Beauveria bassiana*. Therefore, it would not be prudent to include a photo of an insect killed by *Metarhizium anisopliae* outside of the cycle. Additionally, it is quite confusing that the spore colors are shown as white in both cases. This is accurate for *Beauveria bassiana*, but when *Metarhizium anisopliae* causes mycosis in an insect, its spores are olive green.

 In the section on entomopathogenic fungi as bioinsecticides, the authors should briefly mention that entomopathogenic fungi also have the ability to act as endophytes in plants and crops, and that by producing their secondary metabolites, they protect the plants from insect herbivory. This primarily includes fungi like *Beauveria bassiana* and *Metarhizium anisopliae*. This is also a bioinsecticidal activity of these microorganisms.

 Page 13: What are the optimal conditions for the baculoviruses to cause rapid death within 3 to 7 days? This was left ambiguous, so it needs to be clarified.

 Page 17: the numbering of the sections has been lost; it goes from 7.2.2 to 7.3.2. In any case, it should be 7.3. Please review.

Is the review written in accessible language?

Yes

Are all factual statements correct and adequately supported by citations?

Yes

Are the conclusions drawn appropriate in the context of the current research literature?

Yes

Is the topic of the review discussed comprehensively in the context of the current literature?

Partly

Reviewer Expertise:

Biological Control, Integrated Pest Management, Entomology, Microbiology

I confirm that I have read this submission and believe that I have an appropriate level of expertise to confirm that it is of an acceptable scientific standard, however I have significant reservations, as outlined above.

# Response

**Published:** 2024-10-11

**Authors:** Kahsay Tadesse Mawcha

**Affiliations:** Bipesticides, International Centre for Genetic Engineering and Biotechnology, Cape Town, Western Cape, South Africa

Thank you very much for the careful review of our manuscript entitled “ **Recent Advances in Biopesticide Research and Development: A Focus on Microbial: A Review** **. **We appreciate your time and insightful comments on our manuscript.

 We have addressed all the comments and suggestions forwarded from the reviewer. We made significant changes and addressed each point by point as follows:

 Regrads,

 Kahsay

**Introduction section,**

*Page 3, last paragraph. Change Beauveria bassiana to Beauveria spp. since there are many more entomopathogenic species within the Beauveria genus, not just Beauveria bassiana. It would be more accurate to use the genus (Beauveria spp.), just as was done with Metarhizium, Isaria, and Verticillium spp.  *In that same paragraph, the authors should change Verticillium to Lecanicillium according to the current classification of that fungal genus *.*

***= We want to mention the specific Beauveria bassiana. However, we agreed with the reviewer's suggestion and will improve the text to “Metarhizium, Isaria, and Lecanicillium spp.”***

 Page 5. Concepts and classifications of biopesticides. The authors should provide a citation for the idea of biopesticide provided by SADC, just as they did for the concept provided by USEPA. Likewise, they should include a citation for the idea provided by The European Environmental Agency.

 European Environment Agency, 2023. How pesticides impact human health and ecosystems in Europe EEA Briefings 06 (2023)

https://www.sciencedirect.com/science/article/pii/S0269749124005505#bib27

 = ***For the SADC, we provided a link to the reference. We can also give the reference as ….(SADC 2019)***

***SADC Secretariat 2019. SADC (Southern African Development Community) Guidelines for Pesticides Management and Risk Reduction.*** ***http://faolex.fao.org/docs/pdf/sad212166.pdf***

 Page 5: Please provide a citation for the Paris Agreement and the Nagoya Protocol. The authors can find both documents at these links:

https://unfccc.int/sites/default/files/english_paris_agreement.pdf

https://www.cbd.int/abs/doc/protocol/nagoya-protocol-en.pdf

= ***We appreciate the suggestions. We include the citations for******* ***“*** ***The Paris Agreement (United Nations 2015) which is available on the following link.***

*********https://unfccc.int/sites/default/files/english_paris_agreement.pdf***

***For*** ***the Nagoya Protocol 2014. “The Nagoya Protocol on Access to Genetic Resources and The Fair and Equitable Sharing of Benefits Arising from Their Utilization to the Convention on Biological Diversity”*** ***https://www.cbd.int/abs/doc/protocol/nagoya-protocol-en.pdf******* ***Generally, both references can be used in the main text but we may not include them in the reference list as they are not peer-reviewed documents.***

 Page 6: Biopesticide Categories. The authors do not need to repeat the concept of biopesticide again, as this makes the review repetitive and less engaging for the reader, especially considering that a section was already dedicated to detailing the various existing concepts of this term.

**= It is not a repetition. The first one is to show the concepts depending on EPA, EAC, The European Environmental Agency, the International Biocontrol Manufacturer’s Association (IBMA), the International Organization for Biological Control (IOBC), and SADC. The purpose of discussing on page 6 is to demonstrate the different categories of microbial biopesticides NOT all classifications of Biopesticides.**

  Page 11: Figure 3, Mode of Action of Entomopathogenic Fungi. The cycle refers to the infection of *Beauveria bassiana*. Therefore, it would not be prudent to include a photo of an insect killed by *Metarhizium anisopliae* outside of the cycle. Additionally, it is quite confusing that the spore colors are shown as white in both cases. This is accurate for *Beauveria bassiana*, but when *Metarhizium anisopliae* causes mycosis in an insect, its spores are olive green.

***= The color of the spore doesn't matter when it comes to the dead insect, as it doesn't refer to spores when it's killed. The picture aims to illustrate that when microbial biopesticides are used, the target pest being killed is part of the cycle. This is just how it works, and the spores may produce different colors. The purpose of the picture is to demonstrate the mode of action of the entomopathogenic fungi.***

 In the section on entomopathogenic fungi as bioinsecticides, the authors should briefly mention that entomopathogenic fungi also have the ability to act as endophytes in plants and crops, and that by producing their secondary metabolites, they protect the plants from insect herbivory. This primarily includes fungi like *Beauveria bassiana* and *Metarhizium anisopliae*. This is also a bioinsecticidal activity of these microorganisms.

 = ***We have mentioned Beauveria bassiana and Metarhizium anisopliae throughout the text. To avoid repetitions in the review, it is not necessary to include everything in this text. But, if we don’t exceed the page number, we can include the text suggested by the reviewer.***

 Page 13: What are the optimal conditions for the baculoviruses to cause rapid death within 3 to 7 days? This was left ambiguous, so it needs to be clarified.

***=The optimal conditions include optimum temperature and ultraviolet radiation. These have already been mentioned under the advantages of viral biopesticides. The common limitations of all viral products.***

 Page 17: the numbering of the sections has been lost; it goes from 7.2.2 to 7.3.2. In any case, it should be 7.3. Please review.

***=Yes. Accepted. The subtitle “7.3.2 Formulation of fungal biopesticides” should be “7.2.3 Formulation of fungal biopesticides”***

 Many thanks for your time and consideration

 Sincerely,

 Kahsay Tadesse
